# Recent Trends in Bioorthogonal Click-Radiolabeling Reactions Using Fluorine-18

**DOI:** 10.3390/molecules18078618

**Published:** 2013-07-22

**Authors:** Marc Pretze, Doreen Pietzsch, Constantin Mamat

**Affiliations:** 1 Institut für Radiopharmazeutische Krebsforschung, Helmholtz-Zentrum Dresden-Rossendorf, Bautzner Landstraße 400, D-01328 Dresden, Germany; 2 Institut für Klinische Radiologie und Nuklearmedizin, Medizinische Fakultät Mannheim der Universität Heidelberg, Theodor-Kutzer-Ufer 1-3, D-68167 Mannheim, Germany

**Keywords:** Staudinger ligation, Huisgen click reaction, bioorthogonal, radiolabeling, tetrazine

## Abstract

The increasing application of positron emission tomography (PET) in nuclear medicine has stimulated the extensive development of a multitude of novel and versatile bioorthogonal conjugation techniques especially for the radiolabeling of biologically active high molecular weight compounds like peptides, proteins or antibodies. Taking into consideration that the introduction of fluorine-18 (t_1/2_ = 109.8 min) proceeds under harsh conditions, radiolabeling of these biologically active molecules represents an outstanding challenge and is of enormous interest. Special attention has to be paid to the method of ^18^F-introduction. It should proceed in a regioselective manner under mild physiological conditions, in an acceptable time span, with high yields and high specific activities. For these reasons and due to the high number of functional groups found in these compounds, a specific labeling procedure has to be developed for every bioactive macromolecule. Bioorthogonal strategies including the Cu-assisted Huisgen cycloaddition and its copper-free click variant, both Staudinger Ligations or the tetrazine-click reaction have been successfully applied and represent valuable alternatives for the selective introduction of fluorine-18 to overcome the afore mentioned obstacles. This comprehensive review deals with the progress and illustrates the latest developments in the field of bioorthogonal labeling with the focus on the preparation of radiofluorinated building blocks and tracers for molecular imaging.

## 1. Introduction

Positron emission tomography (PET) is an outstanding instrument for the quantification and localization of physiological as well as pathophysiological activities and processes *in vivo* which were analyzed by tracing the appropriate biochemical fundamentals [1]. The basics of PET rely on the coincidental detection of annihilation photons in nearly 180° originating from positrons of the parent radionuclide inside the tracer [2]. Measurements and quantifications of the tracer distribution were accelerated noninvasively in living organisms. For that purpose, fluorine-18 produced by a cyclotron was chosen as the ideal radionuclide due to its favorable half-life. Furthermore, the low positron energy and short ranges in tissues lead to high image resolution [3]. However, tracers for PET imaging purposes are restricted by the kind of fluorinated molecules that researchers can prepare.

In principle, radiolabeling of biologically active compounds and especially high molecular weight compounds like peptides, proteins and antibodies with fluorine-18 still represents a considerable challenge and has therefore generated immense interest. As a result of harsh reaction conditions for the direct introduction of fluorine-18 at high specific activity levels, novel radiolabeling strategies comprising fluorine-18-containing prosthetic groups, also referred as ^18^F-labeling building blocks, are necessary. Due to this fact and due to the multitude of functional groups found in bioactive macromolecules, a labeling strategy has to be developed for almost every one of these compounds. To overcome the above mentioned obstacles, bioorthogonal click-labeling reactions were developed and successfully applied to allow the selective introduction of fluorine-18 under mild conditions, with high radiochemical yields (RCY) and high specific activity (A_S_) [4,5,6,7,8].

The concept of bioorthogonal syntheses refers to all reactions which can proceed inside of living systems without any interference with native biochemical processes. Several requirements have to be fulfilled for a reaction to be considered as bioorthogonal [9]. First and most prominently, selective reactions should be applied to avoid side-reactions with other functional groups found in the biological starting compounds. Both functional groups of the participating reaction partners must be inert to the residual biological moieties and should only react with each other. Secondly, the resulting covalent bond should be strong and chemically inert to biological reactions, and, in addition, it should not affect the (native) biological behavior of the desired molecule. Thirdly, the reaction should benefit from fast reaction kinetics in the case that covalent ligations are achieved prior to probe metabolism and clearance and the reaction must be run on the time scale of cellular processes to prevent biological side reactions. This point is especially important both for the work with short-living positron emitters and further for an application of *in vivo* pre-targeting strategies. At least, the non-toxicity of the reaction partner as well as catalysts are of high importance for *in vivo* applications, and the reaction should proceed under biological conditions taking into account pH, aqueous environments, and temperature.

**Scheme 1:**
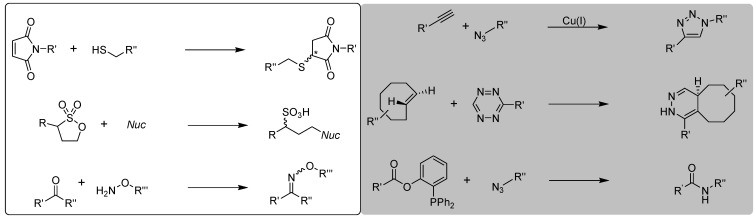
Selected examples of chemical conjugations associated with click chemistry. All reactions highlighted in grey fulfill the bioorthogonality criteria.

Giving consideration to the criteria of bioorthogonal reactions, Kolb, Finn and Sharpless [10,11] defined the click chemistry as a *“set of powerful, highly reliable, and selective reactions for the rapid synthesis of useful new compounds and combinatorial libraries”* and pointed out the following requirements for click reactions: modularity, wideness in scope, very high yielding, stereospecificity (but not necessarily enantioselectivity) and simple product isolation (separation from harmless by-products by non-chromatographic methods). In addition to these criteria, these reactions should proceed using simple reaction conditions (solvent free or solvents like water) including simply accessible starting materials, and the final product has to be stable under physiological conditions [12]. Selected examples of click chemistry reactions are pointed out in Scheme 1.

In recent years, the terms “bioorthogonal reactions” and “click chemistry” have entered into the field of radiochemistry and radiopharmacy. Bioorthogonal reactions in general, and click chemistry in particular, are generic terms for a set of labeling reactions, which make use of several selective and modular building blocks and enable chemoselective ligations to radiolabel biologically relevant compounds. In this context, the Cu(I)-mediated triazole formation from azides and terminal alkynes according to the 1,3-dipolar Huisgen cycloaddition is a particularly powerful ligation reaction, due to its high degree of specificity and the biocompatibility of both starting materials. In the same way, the strain promoted Huisgen click-reaction as well as both variants of the Staudinger Ligation were applied. The absence of catalysts makes these reactions together with the inverse Diels-Alder tetrazine-click reaction tremendously attractive for radiolabeling purposes. Neither azides nor phosphanes, alkenes or tetrazines react with other functional groups commonly present in biopolymers like peptides or proteins. Hence, there is no need for protective group chemistry. Moreover, the desired linking moieties (triazoles, amides or cyclooctapyridazines) are metabolically stable under physiological conditions. As a consequence, click chemistry is a very attractive approach for the design and synthesis of novel potent radiotracers for molecular imaging purposes.

## 2. Fluorine-18 in General

Fluorine-18, with a medium half-life of 109.8 min, is one of 17 known radioisotopes [13] and it was introduced as the most prominent radionuclide for PET imaging [14,15,16,17,18,19,20,21]. The low positron energy (E_β+_ = 0.64 MeV) combined with a short range of the positron in tissue (max. 2.4 mm) provides high resolution images. It can be prepared via cyclotron using the ^18^O(*p*,*n*)^18^F nuclear reaction in a maximum specific activity (A_S_) of 63.3 TBq/µmol no carrier added (n.c.a.) as [^18^F]fluoride or by the ^20^Ne(*d*,α)^18^F nuclear reaction carrier added (c.a.) as [^18/19^]F_2_ [22]. These two species constitute the basis for nucleophilic ([^18^F]fluoride) as well as electrophilic ([^18/19^F]F_2_) labeling reactions. In general, the respective radiotracers were achieved with a lower A_S_ using electrophilic labeling reactions due to the addition of F_2_-gas into the Ne-target [23]. For a successful application of [^18^F]fluoride for radiolabeling purposes, its solubility, nucleophilicity and lipophilicity has to be increased. Standard conditions for the nucleophilic introduction of fluorine-18 generally require: (i) a base such as carbonate, bicarbonate, hydroxide or oxalate, (ii) a phase transfer catalyst such as Kryptofix^®^ K 2.2.2. or tetraalkylammonium salts and finally (iii) a dipolar aprotic solvent such as acetonitrile, DMSO, DMF [24] or alternatively, *tert*-butanol [25].

In most of the cases, radiolabeling with fluorine-18 is achieved by the bioisosterical exchange of a single hydrogen or OH groups. This is due to the fact that fluorine is absent in most biologically active compounds. Only weak steric disturbances occur because fluorine is only slightly larger (1.47 Å) than protons (1.20 Å) and somewhat smaller than oxygen (1.52 Å). Covalently bound fluorine occupies a smaller volume than methyl, amino, or hydroxyl groups, making it favorable for incorporation [26,27]. Next, the resulting C-F bond (453 kJ/mol) is stronger in contrast to the corresponding C-H bond (432 kJ/mol) or C-O bond (378 kJ/mol) [28]. Further, fluorine can also be involved to form hydrogen bonds, but with the difference that fluorine is only a hydrogen bond acceptor compared to a OH group. Notably, a change of the physiological and biological behavior of the fluorine-containing molecule to the desired biological target is often observed in combination of its metabolic stability and lipophilicity.

## 3. Cu-Catalyzed 1,3-Dipolar Huisgen Cycloaddition

1893 was the year of birth of the 1,3-dipolar Huisgen cycloaddition as Arthur Michael described for the first time the preparation of 1,2,3-triazoles from phenylazide and dimethyl but-2-ynedioate [29]. The reaction of hydrogen azide with acetylene to give the corresponding 1,2,3-triazole was first described by Dimroth and Fester 17 years later [30]. Based on this work, Huisgen investigated and specified the character of this reaction as a 1,3-dipolar addition in 1963 [31]. Arbitrary alkynes are used as first reaction partners and serve as dipolarophiles in this reaction. Azides as second reaction partners are ambivalent compounds only describable as zwitterions in an all-octet formula. The driving force for the formation of triazoles in particular is the disappearance of the charges in the azide [32].

**Scheme 2:**
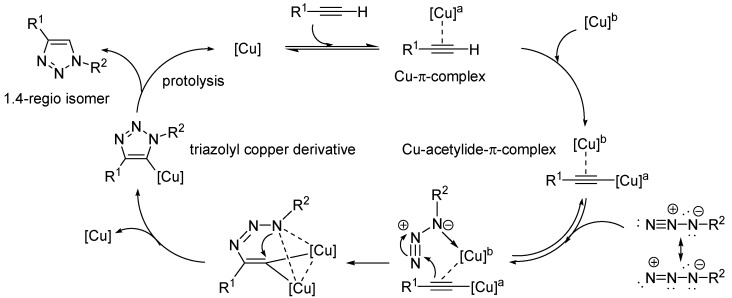
Proposed mechanism of the copper-catalyzed 1,3-dipolar Huisgen cycloaddition (CuAAC) catalyzed by two copper atoms.

Normally, organic azides react with alkynes at high temperatures to give 1,2,3-triazoles in the Huisgen cycloaddition with formation of both 1,4- and 1,5-disubstituted regioisomers. In 2002, the groups of Sharpless *et al.* [33] and Meldal *et al.* [34] independently introduced a revolutionary innovation into the field of 1,3-dipolar Huisgen cycloaddition due to the application of Cu(I)-species as catalyst. The so called **c**opper-catalyzed **a**zide **a**lkyne **c**ycloaddition (CuAAC) proceeds faster, delivers the respective 1,4-isomer exclusively and can be performed at ambient temperatures. Both groups assumed a mechanism in which Cu(I) coordinates first to the terminal alkyne forming a copper(I)acetylide. Computer calculations confirmed these presumptions and predicted a non-concerted mechanism through a six-membered copper(III) metallacycle [35]. Newer findings using continuous-flow analysis cannot exclude a primary addition of the Cu(I) to the azide moiety [36]. Nowadays, Worrel *et al.* have suggested a new mechanism discovered by utilizing ^63^Cu-enriched catalysts. Thus, two copper atoms are incorporated in the catalytic cycle [37] as seen in Scheme 2.

**Scheme 3:**
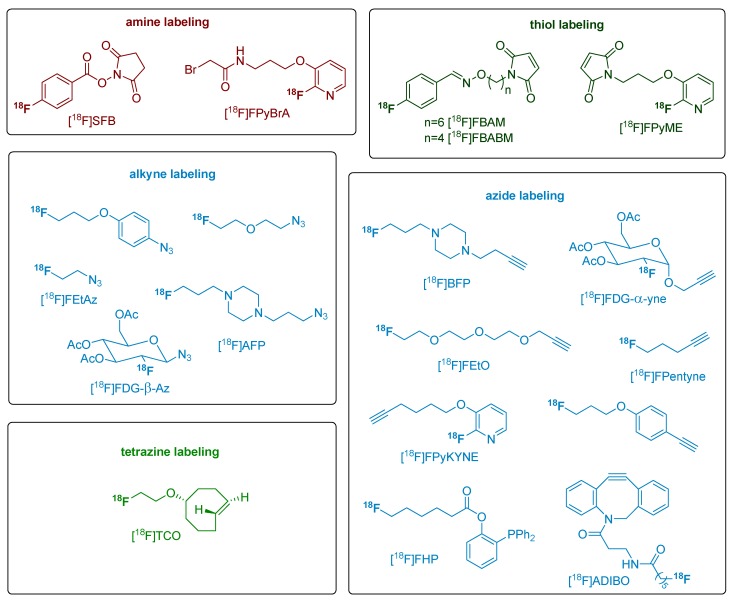
A selection of various conventional and bioorthogonal labeling building blocks for the incorporation of fluorine-18.

As a result of its favorable properties and reaction conditions, the CuAAC has gained enormous popularity and has been applied in diverse fields of chemistry [38]. Since 2006, the CuAAC is introduced for labeling purposes of biologically and pharmacologically relevant molecules with fluorine-18 (Scheme 3). Up until now, over 60 works have been published using this 1,3-dipolar Huisgen cycloaddition for ^18^F-labeling of small organic compounds, peptides, oligonucleotides, proteins and nanoparticles which were partially used for PET studies [5,6,39,40]. The first application of click chemistry for the design and synthesis of radiometal-based radiotracers to form novel multidentate triazole ligand scaffolds for an efficient chelation of the ^99m^Tc(CO)_3_ core was reported by the group of Schibli in 2006 [41]. In 2008, the first application of the CuAAC for the introduction of carbon-11 using [^11^C]methyl azide was published by Schirrmacher and co-workers [42].

The bioorthogonality of both functional groups used in the Huisgen cycloaddition makes this reaction to connect ^18^F-containing building blocks to sensitive bioactive molecules like peptides or proteins highly attractive. Problematically, copper is known to be cytotoxic towards prokaryotic and eukaryotic cells. It is reported that viruses and oligonucleotides are damaged or destroyed in its presence [43]. Other side effects that it is associated with include hepatitis, Alzheimer’s disease or neurological disorders. Therefore, it is of enormous importance for *in vivo* applications to remove the copper completely from the desired radiotracer or to develop copper-free, bioorthogonal alternatives [44].

**Scheme 4:**
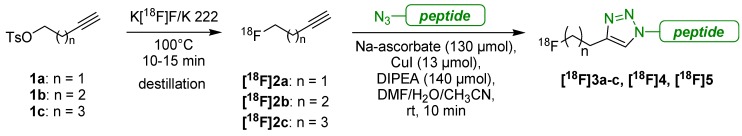
Radiolabeling and distillation procedure followed by the CuAAC according to conditions published by Meldal *et al.* [34].

The first application of the CuAAC in the field of fluorine-18-radiochemistry and -radiopharmacy was published by Marik and Sutcliffe in 2006. They described the synthesis of three ^18^F-containing alkynes **[^18^F]2a–c** with different chain lengths for the radiolabeling of azide-functionalized peptides [45]. For this purpose, three tosylated alkynes **1a–c** were treated with [^18^F]fluoride/K_2_CO_3_/K 222 in acetonitrile at 100 °C (Scheme 4). Due to their fugacity, **[^18^F]2a–c** were purified by co-distillation with acetonitrile into a second vial which was cooled down to −78 °C. It was figured out that the formation of **2b** resulted in the highest radiochemical yield (RCY) of 81% and radiochemical purity (RCP) of 98%. Subsequently, an azide-functionalized model peptide with amino acid sequence YGGFL was radiolabeled with **[^18^F]2a–c** under CuAAC conditions within 10 min. **[^18^F]3b** was obtained with the highest yield (97%) after optimizations. In addition, two other peptides **[^18^F]4** (97%) and **[^18^F]5** (99%) were prepared; the results are shown in Table 1. All peptides were obtained with A_S_ > 35GBq/μmol after isolation using a C18 Sep-Pak extraction. Unreacted building blocks **[^18^F]2a–c** were evaporated together with the eluent to yield all ^18^F-labeled peptides in excellent radiochemical purity. It was also mentioned that the use of bipyridine or bathophenanthroline based ligands for the copper species further improve the yields.

**Table 1:** Results of the radiolabeling with **[^18^F]2a–c**.

Building block	Resulting [^18^F]fluoropeptide	Yield [%]	Purity [%]
**[^18^F]2a**	(^18^F-alkyne, n = 1)-YGGFL **[^18^F]3a**	54	95
**[^18^F]2b**	(^18^F-alkyne, n = 2)-YGGFL **[^18^F]3b**	97	98
**[^18^F]2c**	(^18^F-alkyne, n = 3)-YGGFL **[^18^F]3c**	62	99
**[^18^F]2b**	AGDLHVLR-Ebes-Lys-(^18^F-alkyne, n = 2) **[^18^F]4**	97	81
**[^18^F]2b**	(^18^F-alkyne, n = 2)-AGDLHVLR **[^18^F]5**	99	87

**Scheme 5:**
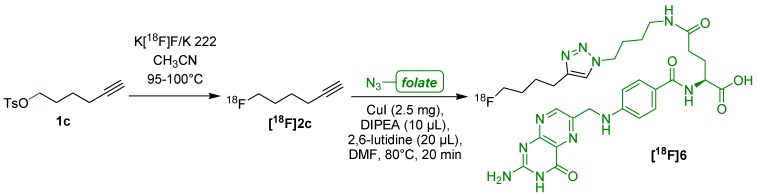
^18^F-labeled folic acid derivative **[^18^F]6**.

Next, Ross *et al.* applied the CuAAC for the synthesis of ^18^F-labeled folic acid derivative **[^18^F]6** using 6-[^18^F]fluorohexyne (**[^18^F]2c**) [46]. After an overall synthesis time of approx. 90 min (Scheme 5), the isolated product **[^18^F]6** was obtained in a RCY of 25–35%, with a RCP > 99% and a A_S_ of 160 ± 70 GBq/μmol. Afterwards, **[^18^F]6** was applied to mice bearing a folate receptor(FR)-expressing KB tumor. Activity accumulation in KB tumors at 45 min p.i. amounted to 3.13% ± 0.83 ID/g. Strong hepatobiliary excretion of the lipophilic tracer led to elevated accumulation (16.53% ID/g) of the activity in the abdominal region.

In 2008, Hausner *et al.* used ^18^F-pentyne **[^18^F]2b** for the radiolabeling of a α_v_β_6_ specific peptide A20FMDV2 [47] and compared it with solid-phase radiolabeling using 4-[^18^F]fluorobenzoic acid [^18^F]FBA as well as 2-[^18^F]fluoropropionic acid [^18^F]FPA. In addition to that case, effects of these labeling moieties on PET imaging and pharmacokinetics were evaluated. In conclusion, the [^18^F]FBA-labeled peptide was obtained in 22 ± 4% RCY and the [^18^F]FPA-incorporated peptide was prepared in 13 ± 3% RCY. In contrast, labeling using **[^18^F]2b** resulted in only 10% RCY for the respective labeled peptide. Therefore, conventional labeling on solid support should be preferred for longer peptides. Additionally, male athymic nude mice bearing α_v_β_6_-positive and α_v_β_6_-negative (control) cell xenografts were employed for biodistribution studies. The click-labeled peptide shows similar tumor uptake but a different metabolism than ulterior ^18^F-labeled peptides.

**Scheme 6:**
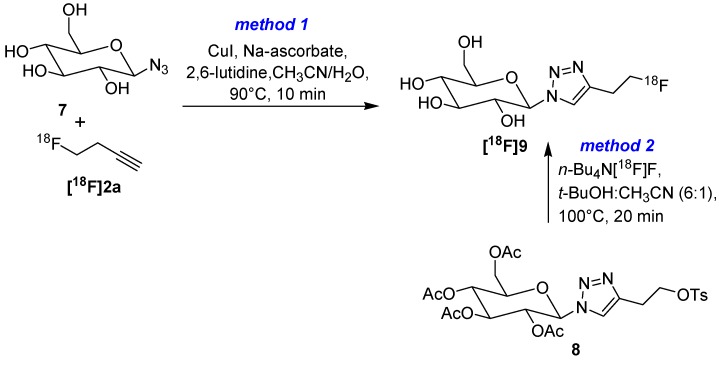
Comparison of conventional nucleophilic labeling with click approach.

In the same year, Kim *et al.* compared a two-step click labeling approach (method 1) with a direct radiofluorination (method 2) for the labeling of β-azido-d-glucose (**7**) as alternative for a radiolabeling with [^18^F]FDG (Scheme 6) [48]. For method 1 (click approach), 4-[^18^F]fluoro-1-butyne (**[^18^F]2a**) was prepared from the appropriate tosylate precursor and was co-distilled into a second vial containing glucopyranosyl azide **7**, CuI, sodium ascorbate and 2,6-lutidine as base. Stirring at 90 °C for 10 min, followed by HPLC purification ensued glucose derivative **[^18^F]9** with a RCY of 30% (d.c.).

Method 2: Tosylate precursor **8** was radiolabeled under conventional conditions with [^18^F]fluoride in a mixture of acetonitrile and *tert*-butanol (v:v = 1:6). **[^18^F]9** was obtained in a RCY of 21% (d.c.) after subsequent removal of acetyl groups with sodium methanolate in methanol and HPLC purification. Surprisingly, the achieved A_S_ for the click approach was higher (59.9 GBq/µmol) compared to the conventional approach (23.5 GBq/µmol). Further, *in vitro* evaluation demonstrated that a triazole moiety at the C1 position of **[^18^F]9** is tolerated neither by the hexokinase nor by the Glut-1 transporter. A slight uptake of **[^18^F]9** in tumor cells (0.2%) could not be blocked.

**Scheme 7:**
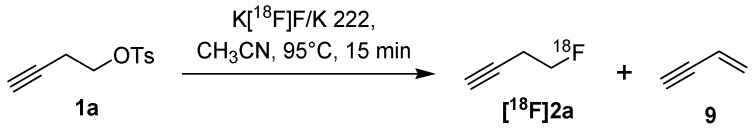
Formation of vinyl acetylene (**9**).

Based on previous studies of Marik and Sutcliffe [45], Kim *et al.* further evaluated 4-[^18^F]fluoro-1-butyne (**[^18^F]2a**) as synthon for ^18^F-click radiolabeling with various azido compounds [49]. They pointed out, that the conditions of the nucleophilic substitution of butyne precursor **1a** with K[^18^F]F delivered a by-product (Scheme 7) in a remarkable percentage which was evidenced as vinyl acetylene (**9**) among the desired ^18^F-butyne **[^18^F]2a** (approx. ratio of **9** and **[^18^F]2a** 79:21 in *t*-BuOH and 87:13 in acetonitrile) while the same reaction using pentyne **1b** exclusively resulted ^18^F-pentyne **[^18^F]2b**. Thus, they underscored the thesis that alkyne-precursors with chain lengths longer than four carbon atoms are better synthons for the preparation of ^18^F-alkyne building blocks due to the suppression of elimination as a side-reaction.

**Scheme 8:**
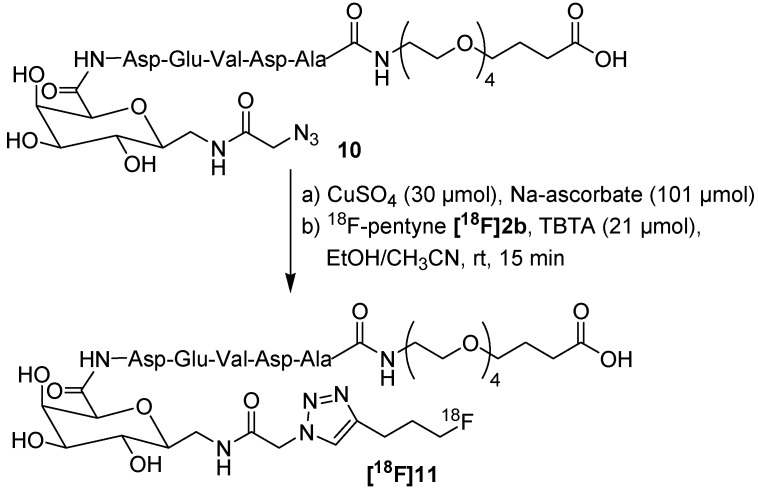
Automated synthesis of ^18^F-labeled peptide **[^18^F]11** for apoptosis imaging.

Later, Zhang *et al.* presented an automated synthesis route for ^18^F-pentyne **[^18^F]2b** which was then applied for ^18^F-labeling of an apoptosis marker (Scheme 8) [50]. **[^18^F]2b** was obtained with a RCY of 51% and was automatically distilled into a second vial containing the peptide precursor **10**. The conversion of ^18^F-pentyne **[^18^F]2b** into ^18^F-peptide **[^18^F]11** amounts nearly 99% after a total synthesis time of 70 min (RCY: 21.0 ± 4.5%, RCP > 99%, A_S_ = 870 GBq/μmol). A549 lung cells were utilized for an *in vitro* evaluation. After treatment with carboplatin, the A549 cell uptake of ^18^F-peptide **[^18^F]11** was found to be 0.86 ± 0.04% together with a cell apoptosis rate of 10.23 ± 2.43%. In contrast, the cell uptake of untreated control cells was found to be 0.06 ± 0.01% (cells apoptosis rate = 1.02 ± 0.31%). From this point of view, ^18^F-peptide **[^18^F]11** could be a promising candidate for future apoptosis imaging.

**Scheme 9:**
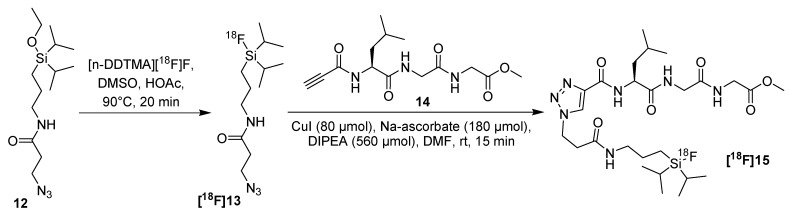
^18^F-labeling of alkyne-functionalized model peptide **14** utilizing ^18^F-Si-based azide **[^18^F]13**.

In 2011, Balentova *et al.* presented an interesting work concerning the ^18^F-labeling of alkyne functionalized model peptide **14** under CuAAC-conditions utilizing a novel and ^18^F-Si-based azide **[^18^F]13** [51]. In general, the high affinity of fluorine for silicon allows a facile introduction of fluorine-18 under mild conditions as well. For this purpose, precursor **12** was prepared and labeled with *n*-dodecyltrimethylammonium [^18^F]fluoride in DMSO at 90 °C for 20 min (RCY 64% for **[^18^F]13**, d.c.). Subsequently, labeling of peptide **14** was accomplished at ambient temperature for 15 min using CuI, sodium ascorbate and DIEA as base. Peptide **[^18^F]15** was obtained in 75% RCY (d.c.) and with a RCP > 97% (Scheme 9).

Problematic, the thermodynamically stable F-Si bond is known to be hydrolyzed in water under physiological conditions unless there are no bulky alkyl groups attached to the silicon. Unfortunately, ^18^F-Si-labeled peptide **[^18^F]15** as well as the building block **[^18^F]13** had a low hydrolytic half-life (<13 min) in buffers at different pH values. The silicon fluorine bond is not enough stabilized by the presence of the two isopropyl groups. 

### 3.1. ^18^Fluoro-PEG_X_-Derivatives

This part of the review deals with PEGylated ^18^F-labeled building blocks. In general, PEGylation describes the process of covalent connection of polyethylene glycol (PEG) moieties to drugs or therapeutic compounds. These compounds are “masked” from the host’s immune system and the water solubility of hydrophobic derivatives is enhanced. Due to the increase of the size its circulatory time is often prolonged by reducing renal clearance.

**Scheme 10:**
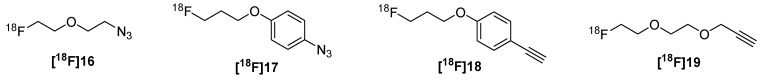
Building blocks evaluated by Sirion and co-workers.

The main advantage of PEGylated ^18^F-labeled building blocks is their reduced volatility compared to the smaller counterparts. Hence, these compounds are easier to handle and the corresponding bioconjugates show a higher blood clearance which is important for *in vivo* studies. In 2007, Sirion *et al.* presented a set of four various ^18^F-labeled building blocks **[^18^F]16** to **[^18^F]19** for the labeling of molecules using the CuAAC (Scheme 10) [52], **[^18^F]19** contains a PEG-like moiety. In the first step, all mesylate precursors were reacted with [^18^F]fluoride in *t*-BuOH at 100 °C for 20 min in the presence of TBAHCO_3_ to obtain the corresponding [^18^F]acetylenes **[^18^F]16** to **[^18^F]19** with RCY ≥ 90%. Further, these building blocks are thermally stable, nearly nonvolatile, and are therefore suitable for one-pot, two-step syntheses.

**Scheme 11:**
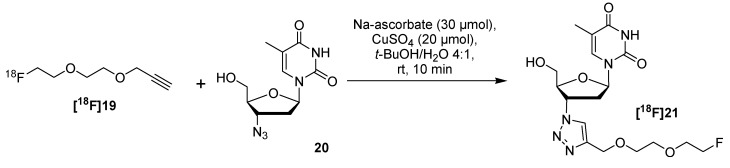
^18^F-labeling of azide functionalized thymidine derivative utilizing **[^18^F]19**.

In the next step, different functionalized biomolecule-like compounds were successfully labeled within 10 min with an excellent ^18^F-conversion of 97–100%. Small non-polar organic compounds needed longer reaction times (30 min) and resulted in limited ^18^F-conversion up to 71%. In most of the cases, the two-step reaction was completed within 40 min from the end of bombardment. The sample labeling reaction of azide functionalized deoxythymidine **20** utilizing alkyne **[^18^F]19** is pointed out in Scheme 11.

**Scheme 12:**
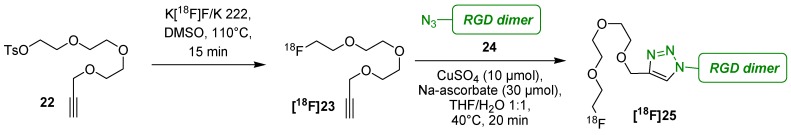
^18^F-labeling of an azide functionalized RGD-dimer utilizing a ^18^F-PEG_3_-alkyne.

Li *et al.* described the preparation of [^18^F]FPTA-RGD_2_ **[^18^F]25** which was labeled with 3-(2-(2-(2-[^18^F]-fluoroethoxy)-ethoxy)ethoxy)prop-1-yne (^18^F-PEG_3_) (**[^18^F]23**) [53]. This labeling building block **[^18^F]23** was obtained in a non decay-corrected yield of 65.0 ± 1.9% from **22**. To increase the affinity the RGD moiety was twice connected to a glutamate which was further functionalized with azide. The major advantage of the click approach was the shortening of the labeling time compared to the conventional labeling with [^18^F]SFB from 180 min to 110 min combined with an increase of the RCY to 53.8% (d.c.) for [^18^F]FPTA-RGD_2_ **[^18^F]25** (Scheme 12). *In vivo* evaluations of **[^18^F]25** using athymic nude mice bearing a U87MG tumor demonstrated a lower α_v_β_3_-integrin affinity in contrast to other ^18^F-labeled RGD peptides due to the relatively long PEG_3_ moiety.

In 2009, the group of Devaraj *et al.* used the same ^18^F-building block ^18^F-PEG_3_ **[^18^F]23** for the preparation of ^18^F-CLIO – a fluorine-18-containing trimodal nanoparticle [54]. The basis of this particle consists of an aminated cross-linked dextran iron oxide (CLIO) decorated with diethylene triamine pentaacetic acid. The advantage of ^18^F-CLIO was the ability of a triple detection by PET, fluorescence molecular tomography as well as magnetic resonance imaging. The ^18^F-nanoparticle was prepared as briefly follows: ^18^F-PEG_3_ **[^18^F]23** was added to the azido-CLIO in a PBS buffer. This mixture was then treated with CuSO_4_/bathophenanthrolinedisulfonic acid (BPDS, as disodium salt) and sodium ascorbate in distilled water. The reaction mixture was incubated for 40 min at 40 °C and filtrated to remove the resulting ^18^F-CLIO from unreacted ^18^F-PEG_3_ with a RCY of 58% (d.c.).

First evaluations of ^18^F-CLIO suspended in the agar phantom pointed out a detection threshold of 0.025 µg Fe/mL for PET-CT imaging. This is approximately 200 times lower than by T2*-weighted MR imaging. The lowest observed concentration of the nanoparticles by MRI was 5 µg Fe/mL (15), demonstrating a PET detection threshold 200 times more sensitive than MRI at current specific activities. A high *in vivo* stability for ^18^F-CLIO was demonstrated due to the low renal and bladder uptake, no excretion via the kidneys was observed.

**Scheme 13:**
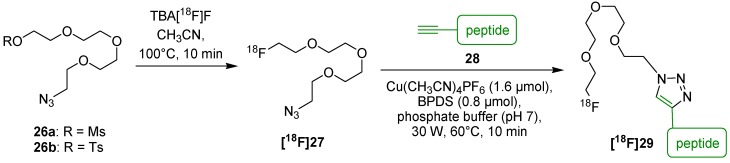
^18^F-labeling of an alkyne functionalized peptide **28** utilizing ^18^F-PEG_3_-azide **[^18^F]27**.

In 2011, Gill and Marik used optimized conditions and switched the bioorthogonal functionalities for radiolabeling of *N*-alkynylated peptide **28** (4-pentynoyl-Val-Asp-Asn-Lys-Phe-Asn-Lys-Glu-Nle-Gln-Asn-Ala-Tyr-Ala-Ile-Glu-Ile-Ala-Leu-Leu-Pro-Asn-NH_2_) using ^18^F-PEG_3_-azide **[^18^F]27** (Scheme 13) [55]. After purification by HPLC, ^18^F-labeled peptide **[^18^F]29** could be obtained with RCY of 31% and a A_S_ = 1.44 GBq/µmol. The removal of BPDS was difficult using solid phase extraction (SPE) which lowered the chemical purity. Therefore, optimized reaction conditions without BPDS provided 96% conversion after 60 °C for 10 min when using 1.6 µmol Cu(CH_3_CN)_4_PF_6_ and 10 nmol of **28** at high concentrations (125 µL). Noteworthy, a maximum A_S_ and RCP were only obtainable when **28** was quantitatively converted to ^18^F-labeled peptide **[^18^F]29** due to their similar molar mass and retention times in HPLC and SPE. Furthermore, the copper source should be considered very crucial, since PF_6_^-^ can lower the A_S_ due to an isotopic exchange.

In 2012, Lee *et al.* used the same ^18^F-PEG_3_-azide **[^18^F]27** for the radiolabeling of ZnO nanoparticles (20 nm) in order to observe their behavior and accumulation in organic tissues after oral administration using PET [56]. ^18^F-labeling of ZnO particles resulted in A_S_ = 0.73 MBq/mg and RCP > 95%. PET images indicated that ^18^F and ^18^F-ethoxyazide showed radioactivity in the bone and bladder 3 h after oral administration, whereas radioactivity for ^18^F-labeled ZnO nanoparticles was seen only in the gastrointestinal (GI) tract. At 5 h post-administration, biodistribution studies demonstrate that ^18^F-labeled ZnO nanoparticles showed radioactivity in the lung, liver and kidney including the GI tract.

### 3.2. [^18^F]Fluoroethylazide

**Scheme 14:**
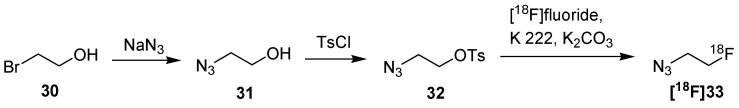
Reaction sequence for the preparation of (^18^F-FEA) **[^18^F]33**.

[^18^F]Fluoroethylazide–^18^F-FEA (**[^18^F]33**) is certainly the most popular building block for radiolabeling purposes using the CuAAC. The first approach was evaluated and applied by Glaser and Årstad in 2007 [57]. 2-Azidoethyl-4-toluenesulfonate (**32**) was used as starting material and the subsequent nucleophilic fluorination with anhydrous n.c.a. K 222, K[^18^F]F in acetonitrile at 80 °C for 15 min yielded the desired azide **[^18^F]33** (RCY = 55%, d.c.). The isolation was done by distillation. The reaction sequence for ^18^F-FEA **[^18^F]33** is pointed out in Scheme 14.

In most of the cases, the isolation of **[^18^F]33** was accomplished by co-distillation with acetonitrile. Notably, tosylate **32** is also co-distilled when using higher temperatures (>90 °C), and **32** reacts with the alkyne precursor as well in the subsequent labeling step. At temperatures below 90 °C the transfer of tosylate **32** to the cooled receiver vial was negligible, but the distillation time had to be extended [58]. Next, the somewhat difficult handling due to the volatility of the building block during the radiosynthesis brought different reaction set-ups in order to make the handling of ^18^F-FEA **[^18^F]33** easier. Up to now, the [^18^F]FEA-synthesis and the following labeling step with the respective alkyne were transferred into a remotely controlled module using a cooling trap [58].

**Scheme 15:**
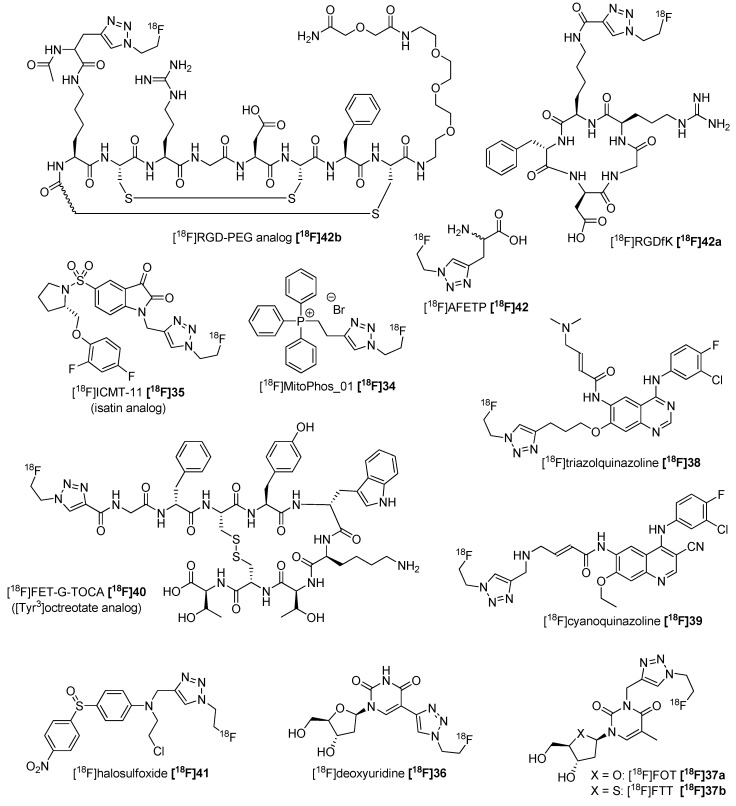
Overview of radiotracers prepared by CuAAC with ^18^F-FEA **[^18^F]33**.

The convenient and fast access of ^18^F-FEA **[^18^F]33** led to multitude of radiotracers, e.g., for the imaging of apoptosis with [^18^F]MitoPhos_01 **[^18^F]34** [59] or isatine derivatives like [^18^F]ICMT-11 **[^18^F]35** [60,61] connected with a GMP-applicable automated synthesis [62]. Furthermore, tracers for proliferation like [^18^F]deoxyuridine **[^18^F]36** [63] or [^18^F]FOT and [^18^F]FTT **[^18^F]37a,b** [64] were developed. ^18^F-labeled quinazoline derivatives **[^18^F]38** and **[^18^F]39** for the EGFR activity imaging were prepared [58,65]. [^18^F]FET-G-TOCA **[^18^F]40** for the somatostatin receptor [66], [^18^F]haloethylsulfoxides like **[^18^F]41** [67] and six ^18^F-labeled nitroaromates based on [^18^F]MISO [68] to visualize hypoxia were evaluated. Further, it is possible to address the α_v_β_3_ receptor with ^18^F-containing RGD peptides e.g., **[^18^F]42a,b** [69,70,71]. [^18^F]AFETP **[^18^F]43** was introduced as a tracer for the imaging of brain tumors [72] and mouse DBT glioma [73]. Galante *et al.* used ^18^F-FEA **[^18^F]33** for the radiofluorination of a series of 6-halopurines in a one-pot automated synthesis approach [74]. They used the copper chelator bathophenanthrolinedisulfonate to accelerate the cycloaddition reaction. An overview of all these tracers is given in Scheme 15.

**Scheme 16:**
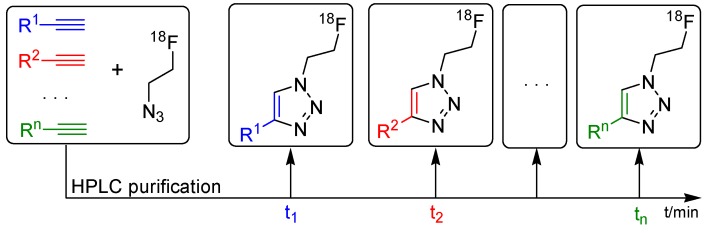
The technical route of the multiple labeling procedure.

The last approach using [^18^F]fluoroethylazide (**[^18^F]33**) was published by Jia *et al.* in 2013 [75]. This group demonstrated the simultaneous labeling of various alkynes with ^18^F-FEA **[^18^F]33** in a one-pot procedure. In general, two or more different alkynes were mixed in a DMF-solution together with CuSO_4_ and sodium ascorbate. Afterwards, a solution of **[^18^F]33** in acetonitrile was added, the resulting mixture was shaken at 60 °C for 15 min and then analyzed/purified via HPLC after filtration. This procedure is pointed out in Scheme 16. Importantly, an appropriate HPLC separation system has to be developed for the separation of the resulting tracers prior the radiolabeling.

In 2012 Glaser *et al.* reported, that ^18^F-FEA **[^18^F]33** is able to be reduced to give ^18^F-fluoroethylamine by the use of copper wire under acidic conditions, which could be a reason for the sometimes low RCY in click reactions [76]. Another approach published by Zhou *et al.* in 2012 used ^18^F-FEA **[^18^F]33** for the synthesis of a [^18^F]SFB analog [77]. They pointed out that base labile NHS-esters tolerate the “click” conditions well. Moreover, the resulting compound can be prepared in a shorter synthesis time (~ 35 min) in contrast to [^18^F]SFB (70 min) and in a higher RCY.

**Scheme 17:**
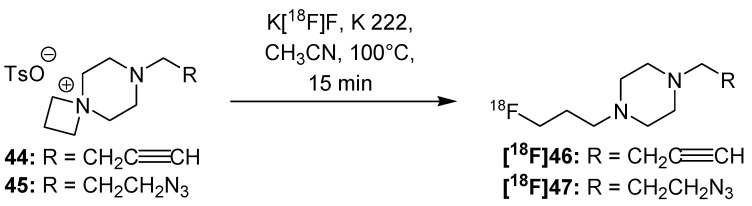
Preparation of BFP **[^18^F]46** and AFP **[^18^F]47**.

Two novel and universally applicable building blocks BFP **[^18^F]46** and AFP **[^18^F]47** were prepared by Pretze *et al.* in 2013 (Scheme 17) [78]. In addition to the Huisgen-click reaction, it is also possible to apply the Staudinger Ligation as well for radiolabeling purposes when using [^18^F]AFP **[^18^F]47**. Furthermore, an automated module synthesis was evaluated for both building blocks. The introduction of spiro salts **44** and **45** as precursors led to a simplified separation and purification from the resulting building blocks via elution using RP18 or Si cartridges in a short timespan.

**Scheme 18:**
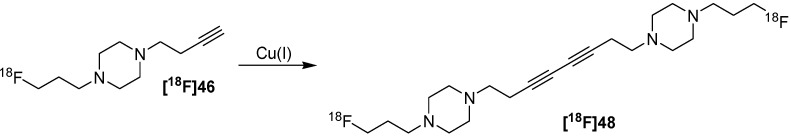
Glaser coupling with [^18^F]BFP **[^18^F]46** as side reaction.

**Scheme 19:**
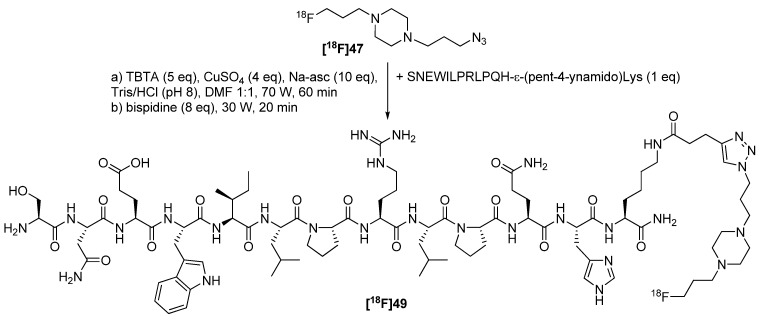
Radiolabeling of a SNEW peptide using [^18^F]AFP **[^18^F]47**.

Both building blocks **[^18^F]46** and **[^18^F]47** were used for the radiolabeling of azide as well as alkyne functionalized peptides with SNEW sequence for an imaging of the EphB2 receptor [79]. Problematically, Glaser coupling was observed as a side reaction using [^18^F]BFP **[^18^F]46** for the labeling of peptides (Scheme 18). To overcome this fact, [^18^F]AFP **[^18^F]47** was used instead and the labeling procedure was accomplished on resin (Scheme 19). A second important fact is the removal of the copper. It was found that the copper forms strong complexes with this kind of peptides. For this reason, Cu-containing peptides do not behave like “normal” peptides. To fix this obstacle, bispidine [80], a strong chelating agent, was used to wash the resin after the click reaction, while other chelating agents failed.

### 3.3. ^18^Fluoro-Aryl Building Blocks

**Scheme 20:**
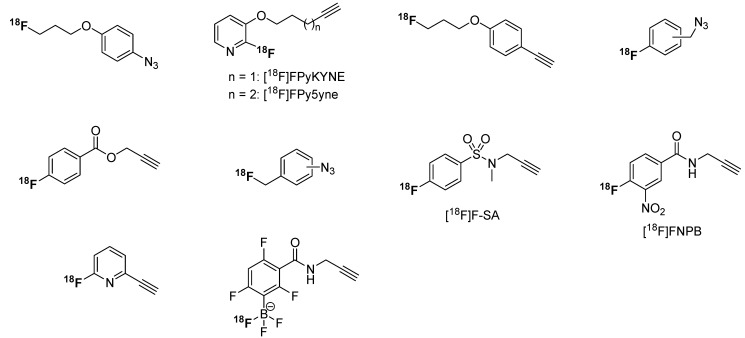
Overview of [^18^F]fluoroaryl building blocks.

The ^18^F-labeling of aryl precursors is a common task for radiochemists. In general, fluoroaryl compounds possess a higher metabolic stability *in vivo* than fluoroalkyl moieties due to the fluorine bound to the sp^2^-carbon atom. In most cases, the radiolabeled products show a higher lipophilicity due to the aryl moiety and therefore play a key role in the design of lipophilic radiotracers. A multitude of different aryl click building blocks were evaluated in the past. An overview is shown in Scheme 20.

The first approach for the radiofluorination of biomolecules utilizing the sulfonamide-based alkyne 4-[^18^F]fluoro-*N*-(prop-2-ynyl)benzamide–[^18^F]F-SA (**[^18^F]51**) as ^18^F-labeling building block was presented by Ramenda *et al.* in 2007 [81]. [^18^F]F-SA **[^18^F]51** was synthesized within 80 min including HPLC purification with RCY of 32 ± 5% (RCP > 95%, A_S_ = 120–570 GBq/µmol) in a remotely controlled module. Next, **[^18^F]51** was applied for the radiolabeling of an azide functionalized neurotensin(8-13); the labeled peptide was obtained with a RCY = 66% as determined by radio-HPLC. The *in vitro* binding affinity (IC_50_) of ^18^F-neurotensin(8-13) derivative was determined to be 66 nM. 

**Scheme 21:**
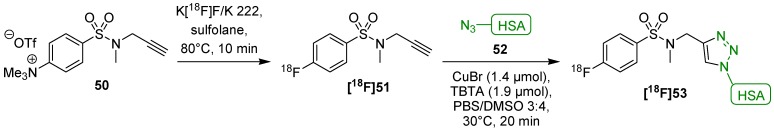
Preparation of **[^18^F]51** and subsequent radiolabeling of HSA **52**.

Furthermore, the same building block [^18^F]F-SA **[^18^F]51** was applied for ^18^F-labeling of azide-functionalized human serum albumin (HSA) **52** [82]. ^18^F-Labeled HSA **[^18^F]53** was purified via size-exclusion chromatography (SEC) and was obtained with a RCY of 55% in a total synthesis time of 120 min starting from [^18^F]fluoride (Scheme 21). Finally, metabolite analyses of ^18^F-HSA **[^18^F]53** were accomplished using nude mice. After 120 min, most of the activity was found in the gall bladder, intestines and in the bladder. The half-life of blood activity of **[^18^F]53** was calculated to be 31 min. Further, the introduction of the azide residue into HSA and subsequent the radiolabeling has significantly altered the structural and functional integrity of HSA.

In 2013, the same group extended their repertoire and presented the radiolabeling of an azide-modified phosphopeptide (H-Met-Gln-Ser-pThr-Pro-Leu-OH) and an l-oligonucleotide (17-mer l-RNA: 5’-CCGCACCGCACAGCCGC-3’) with [^18^F]F-SA **[^18^F]51** [83]. They pointed out that the lipophilicity (logP 1.7) of [^18^F]F-SA **[^18^F]51** allows radiolabeling in aqueous solutions. After the click reaction, the ^18^F-phosphopeptide was obtained in 73 to 77% RCY at low precursor amounts (100–400 µg). Additionally, the ^18^F-labeled oligonucleotide was isolated in 44% RCY as determined by radio-HPLC after 20 min click-reaction time.

**Scheme 22:**
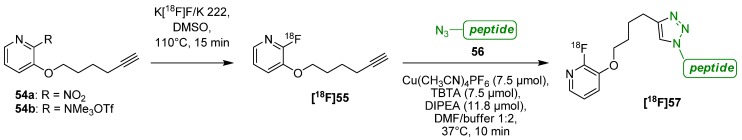
Radiosynthesis of [^18^F]FPy5yne **[^18^F]55** and CuAAC with model peptide **56**.

The first approach for radiolabeling with pyridine-based ^18^F-heteroaryl building blocks was presented by Inkster *et al.* in 2008 [84]. For this purpose, 2-[^18^F]fluoro-3-(hex-5-ynyloxy)pyridine–[^18^F]FPy5yne (**[^18^F]55**) was prepared via nucleophilic (hetero-)aromatic substitution from either the corresponding 2-nitro or 2-trimethylammonium pyridine **54a,b**. Both precursors delivered **[^18^F]55** in a high RCY of > 90% after 15 min synthesis time and 110 °C in DMSO or acetonitrile. Further, model peptide N_3_-(CH_2_)_4_-CO-Tyr-Lys-Arg-Ile-OH **56** was treated with **[^18^F]55** under click-conditions to obtain **[^18^F]57** in a RCY of 18.7% (d.c.) within a total preparation time of 160 min (start of synthesis). Tris(benzyltriazolylmethyl)amine (TBTA) was used as stabilizing agent for Cu(I). Results are summarized in Scheme 22.

Furthermore, [^18^F]FPy5yne **[^18^F]55** was applied for the first radiofluorination of an azide-functionalized DNA analog [85]. For that reason, a 5’-azide-modified DNA sequence antisense to *mdr1* mRNA was prepared and treated with **[^18^F]55** in the presence of TBTA and 2,6-lutidine instead of DIPEA. ^18^F-Labeled DNA sequence was isolated with 24.6% ± 0.5% RCY (d.c., after EOB) in a shortest preparation time of 276 min from start of synthesis. The copper source was CuBr (99.9999% purity). The DNA sequence antisense to mRNA transcribed by the *mdr1* gene could be used for imaging of breast cancer.

Moreover, the group of Valdivia *et al.* presented an approach for the synthesis of 2-[^18^F]fluoro-3-pent-4-yn-1-yloxypyridine–[^18^F]FPyKYNE which is one CH_2_ group shorter than the aforementioned [^18^F]FPy5yne **[^18^F]55**. An automated synthesis procedure was evaluated for [^18^F]FPyKYNE and the subsequent radiolabeling of an RGD peptide. The labeled peptide was obtained in a total RCY of 12–18% with a RCP > 99% after 125 min in a two-step, two-reactor process [86].

**Scheme 23:**
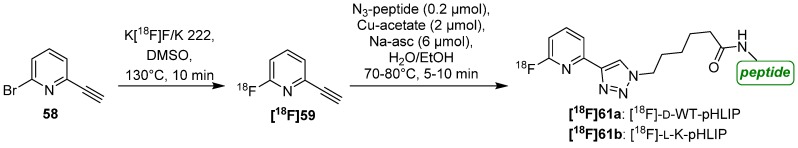
^18^F-labeling procedure for two pHLIP peptide analogs **[^18^F]61a** and **[^18^F]61b**.

Finally, 6-[^18^F]fluoro-2-ethynylpyridine **[^18^F]59** was prepared as the third heteroaromatic example from the corresponding bromo precursor **58** within 10 min at 130°C with a RCY of 27.5 ± 6.6% (d.c.) with high RCP ≥ 98% by Daumar *et al.* in 2012 [87]. A d-amino acid analogue of WT-pHLIP and an l-amino acid peptide K-pHLIP were used, both functionalized at the *N*-terminus with 6-azidohexanoic acid. The subsequent click-labeling was performed at 70 °C in a mixture of water and ethanol using Cu-acetate and sodium ascorbate (Scheme 23). [^18^F]-d-WT-pHLIP **[^18^F]61a** and [^18^F]-l-K-pHLIP **[^18^F]61b** were obtained with RCYs between 5−20% after HPLC purification in a total reaction time of 85 min including the formulation for the biological evaluation. *In vitro* stability tests in human and mouse plasma revealed high stability. After 120 min, 65% of **[^18^F]61a** and 85% of **[^18^F]61b** remained intact. PET imaging and biodistribution studies in LNCaP and PC-3 xenografted mice revealed pH-dependent tumor retention.

**Scheme 24:**
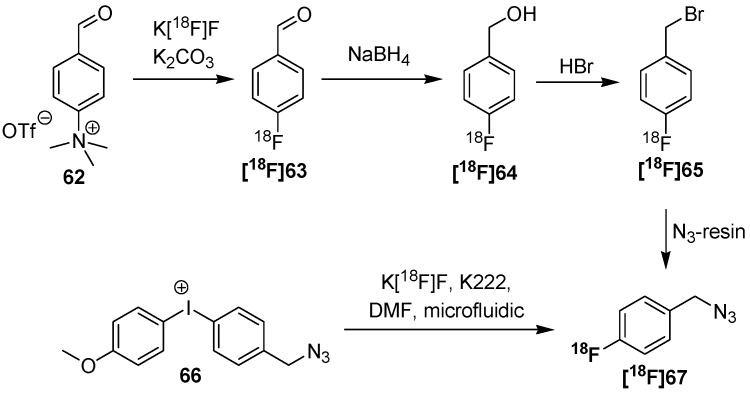
Comparison between labeling of trimethylammonium precursors and diaryliodonium salts.

Next, two approaches for the preparation of (azidomethyl)-[^18^F]fluorobenzenes like **[^18^F]67** were presented by Thonon *et al.* [88] and Chun *et al.* [89] (Scheme 24). The first group used a four-step synthesis path starting from 4-trimethylammonium benzaldehyde (**62**). Fluorine-18 was introduced in the first step followed by reduction of the aldehyde **[^18^F]63**, conversion into the bromo compound **[^18^F]65** and final functionalization with azide. 34% RCY (d.c.) of **[^18^F]67** could be obtained within 75 min starting from [^18^F]fluoride. The second group used a single step strategy. Thus, diaryliodonium salts like **66** were introduced as precursor. Electron-rich aryl moieties, such as a 4-methoxyphenyl or 2-thienyl, are generally known to direct the [^18^F]fluoride into the other relatively electron-deficient ring of this diaryliodonium salt [90]. Each precursor was subjected to labeling with [^18^F]fluoride (n.c.a.) in the microfluidic device. The respective building blocks were obtained after 94−188 s in high RCY’s (35−45% for **[^18^F]67**) (d.c.) whereas the by-product was only formed in <3%.

**Scheme 25:**
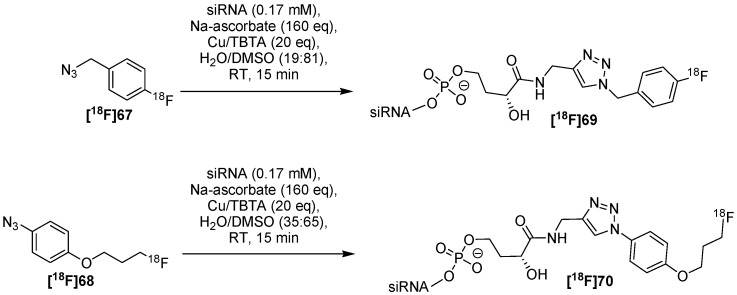
Labeling procedure of siRNA **[^18^F]69** and **[^18^F]70**.

The group of Thonon *et al.* [88] used **[^18^F]67** for subsequent click labeling of a 4-ethynyl-l-phenylalanine-containing peptide. For this purpose, the click-conditions were optimized and the ligation reaction was finished in less than 15 min. Later in 2011, the same group presented a general method for the labeling of siRNA [91]. Two complementary building blocks 1-(azidomethyl)-4-[^18^F]fluorobenzene **[^18^F]67** and 1-azido-4-(3-[^18^F]fluoropropoxy)benzene **[^18^F]68** have been produced with 41% and 35% RCY (d.c.), respectively. The whole labeling procedure takes 120 min with a RCY of 15 ± 5% (d.c.) for both pure [^18^F]siRNA derivatives **[^18^F]69** and **[^18^F]70** (Scheme 25).

In 2012, Flagothier *et al.* investigated the influence of the linkers connected to the oligonucleotides and presented a new alkyne-functionalized linker for siRNA [92]. For subsequent labeling purposes, **[^18^F]67** was prepared in 45 min with 84% RCY using a remotely controlled device (GE Fastlab^®^). The siRNA concentration was used in a low concentration of about 10^-5^ M and represents an important advantage of the method considering the high cost of this precursor. [^18^F]siRNA was prepared under the same conditions as pointed out by Mercier *et al.* [91] in a comparable RCY of 12%.

Next, two further labeling building blocks based on the 4-[^18^F]fluorobenzoate skeleton were developed. First, propargyl 4-[^18^F]fluorobenzoate–[^18^F]PFB was prepared by the group of Vaidyanathan *et al.* from the respective trimethylammonium precursor in a RCY of 58 ± 31% (d.c.) within 15 min. Afterwards, several model compounds and a transglutaminase-reactive peptide (RCY = 37 ± 31%) were labeled using [^18^F]PFB [93]. The second approach consists of the preparation of 4-[^18^F]fluoro-3-nitro-*N*-2-propyn-1-yl-benzamide – [^18^F]FNPB published by Li *et al.* in 2012 [94]. [^18^F]FNPB was synthesized with a RCY of 58% (A_S_ > 350 GBq/µmol, RCP > 98%) within 40 min. *In vitro* stability tests using mouse plasma revealed no radiodefluorination over 2 h. Afterwards, three different azide-modified peptides were labeled with RCYs between 87 and 93%.

**Scheme 26:**
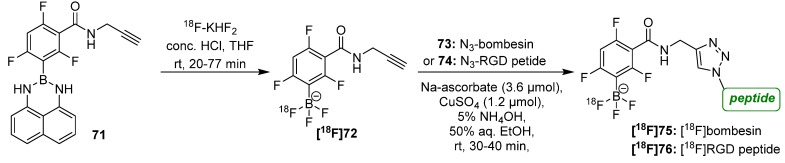
Arylboronate **[^18^F]72** used for radiofluorination purposes.

Further, an unconventional and promising approach was introduced by Li *et al.* in 2013 consisting of a radiolabeling procedure using alkyne functionalized aryltrifluoroborate **[^18^F]72** and either azide-modified bombesin **73** or RGD peptide **74** [95,96]. Advantageously, arylboronates like **71** capture [^18^F]fluoride directly and rapidly under aqueous conditions (20 min, 20–40°C, pH 2–3, then quench to pH 7.5) under formation of a water-soluble, non-coordinating aryltrifluoroborate anion **[^18^F]72** that is highly polar (logP < −4). The alkyne-[^18^F]ArBF_3_^−^ **[^18^F]72** was subsequently conjugated to both peptides within 25 min without need for prior work-up in this two-step one-pot procedure (Scheme 26).

Later, the specific activity for the building block as well as for the labeled peptides was improved. This was possible due to the stoichiometry of the introduction of n.c.a. [^18^F]fluoride into the precursor. Investigations to determine the A_S_ were done using a rhodamine-N_3_ conjugate. After this improvement, it was possible to prepare alkyne-[^18^F]ArBF_3_^−^ **[^18^F]72** with a A_S_ = 148 GBq/μmol and the resulting RGD peptide **[^18^F]76** with a A_S_ = 444 GBq/μmol. The tumor-uptake and corresponding good PET imaging of both peptides was accompanied by a three times higher A_S_ which was statistically verified [97].

### 3.4. ^18^Fluoro-Gluco-Derivatives

The glycosylation of peptides represents an important tool for the enhancement of their *in vivo* behavior like blood clearance and stability. For this reason, labeling building blocks based on carbohydrates for the glycosylation of potential radiotracers using the CuAAC were evaluated by Maschauer and Prante in 2009 [98]. In this context, different α- and β-anomeric azides and alkynes were designed for radiolabeling purposes. They demonstrated that the radiolabeling of triflated precursors with fluorine-18 proceed better when using β-anomeric derivatives like **77** (RCY of **[^18^F]78** was 71 ± 10%). Afterwards, a proof-of-principle reaction of the ^18^F-building block **[^18^F]78** was accomplished by labeling Fmoc-l-propargylglycine with a RCY of 60% for unprotected building block and a RCY of 76% for the peracetylated building block.

**Scheme 27:**
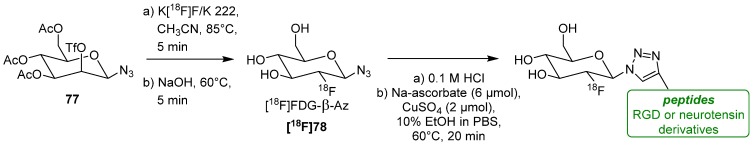
Preparation of mannopyranosyl azide **77** and subsequent ^18^F-glycosylation with **[^18^F]78**.

Finally in 2010, they applied **[^18^F]78** as the building block with the highest RCY to label different peptide moieties like RGD or neurotensin derivatized with l-propargylglycine (Scheme 27). RCYs ranged from 17 to 20% and A_S_ = 55–210 GBq/µmol were obtained for the labeled conjugates in a total synthesis time of 75 min [99]. Furthermore, *in vivo* investigations with the [^18^F]FGlc-RGD peptide were carried out using U87MG-bearing mice. Because of the glycosylation they had a better blood clearance and stability in α_v_β_3_-integrin expressing tumors. The specific tumor uptake of [^18^F]FGlc-RGD was 0.49% ID/g (60 min p.i.).

**Scheme 28:**
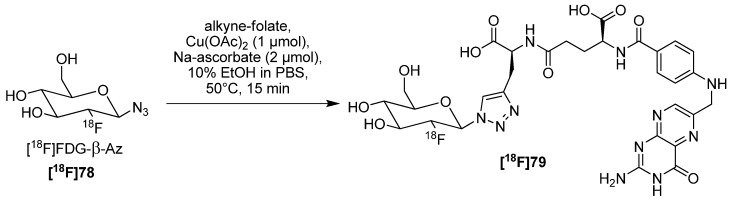
^18^F-glycosylation of a folate derivative.

In 2012, Fischer *et al.* adapted this method to label a novel folic acid conjugate in order to image the folate receptor, which is overexpressed in various tumor entities [100]. After 3 h of synthesis, [^18^F]fluorodeoxyglucosyl folate **[^18^F]79** was obtained in RCY = 5−25% (d.c.), with a A_S_ = 90 ± 38 GBq/μmol and a RCP > 95% (Scheme 28). The biodistribution and PET imaging studies in KB tumor-bearing mice showed a high and specific uptake of the radiotracer in FR-positive tumors (10.03 ± 1.12% ID/g, 60 min p.i.) and kidneys (42.94 ± 2.04% ID/g, 60 min p.i.).

## 4. Strain-Promoted Huisgen Cycloaddition

Strain-promoted and copper-free variants of the Huisgen cycloaddition of cyclic alkynes with azides are of great interest as powerful tools in reagent/catalyst-free bioconjugations. This type of reaction was applied especially for labeling purposes on living cells or *in vivo* studies due to the mild reaction conditions and the bioorthogonal character expressed by a high specificity and selectivity. Advantageously, the use of cytotoxic Cu(I) was not mandatory in contrast to the CuAAC. Cyclooctyne is the smallest stable cyclic alkyne and has a high reaction potential to react with azides as demonstrated by Blomquist and Liu in 1953 [101] and by Wittig and Krebs in 1961 [102]. The first connection of this type of click-reaction with cyclooctyne connected with a biological application was recognized by Agard *et al.* in 2004 [103].

With the entry of copper-free 1,3-dipolar cycloaddition reactions in radiopharmacy, several strain-promoted systems, such as cyclooctynes and dibenzocyclooctynes, have been developed for the incorporation of fluorine-18. Additionally, a few examples of radiometal labeling were reported [104].

In general, two strategies were pointed out. The first comprises the preparation of fluorine-18-containing cyclooctyne derivatives which were applied for radiolabeling of azide-functionalized molecules of interest. Alongside, ^18^F-azide building blocks were synthesized and used for the labeling of cyclooctyne-bearing bioactive compounds. In general, two regioisomers are obtained in both strategies of this reaction which could indicate a different biological behavior in certain circumstances depending on the remaining molecule residue.

**Scheme 29:**
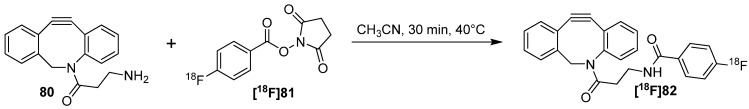
[^18^F]Fluorobenzoate-functionalization of DBCO **80**.

The first example of a biological application in combination with ^18^F-radiolabeling was published in 2011 by Bouvet *et al.* [105]. Thus, a fluorine-18-labeled aza-dibenzocyclooctyne (DBCO) building block **[^18^F]82** was synthesized through acylation of commercially available *N*-(3-amino-propionyl)-5,6-dihydro-11,12-didehydrodibenzo-[*b*,*f*]azocine (**80**) with *N*-succinimidyl 4-[^18^F]fluoro-benzoate–[^18^F]SFB (**[^18^F]81**) (Scheme 29). For this purpose, amino compound **80** and [^18^F]SFB **[^18^F]81** were dissolved in acetonitrile and reacted for 30 min at 40 °C monitored by radio-HPLC. After separation using semipreparative radio-HPLC, cyclooctyne **[^18^F]82** was obtained with a RCY of 85% and a RCP > 95%. To determine the *in vivo* stability of **[^18^F]82**, metabolite analyses was performed using Balb/C mice. After 60 min p.i., 60% of compound **[^18^F]82** was still intact. Further, dynamic small animal PET studies showed rapid clearance of [^18^F]FB-DBCO **[^18^F]82** from the blood (blood clearance half-life: 53 s) and from most other tissues and organs. Most activity was found in the bladder, gall bladder and intestines.

**Scheme 30:**
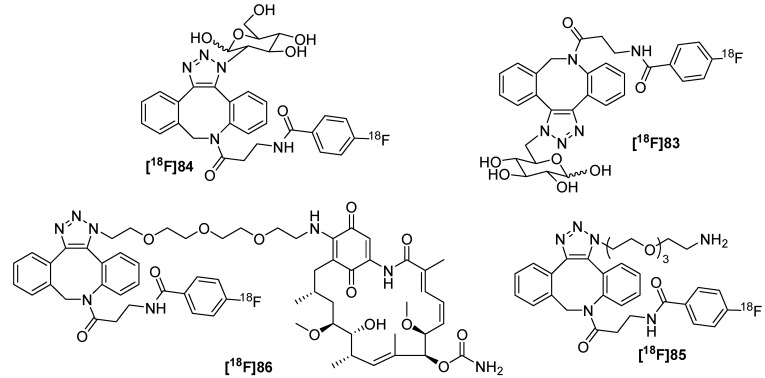
Scope of the labeling reaction with **[^18^F]80**.

Subsequently, various azide-functionalized sample molecules and bioactive compounds of interest like carbohydrates and geldanamycine were conjugated and radiolabeled with **[^18^F]82** (for an overview see Scheme 30). For the preparation of the non-radioactive references, 1.5 to 2.0 equiv. of respective azides were treated with 1 equiv. of FB-DBCO. All reactions resulted in the formation of two distinct regioisomers (1,4- and 1,5-triazole regioisomer). Products were isolated using HPLC-purification; but in some cases both regioisomers were not separable. Five different reaction conditions were tested for the radiolabeling procedure with **[^18^F]82** (a: methanol, room temperature, 15 min; b: phosphate buffer, 40 °C, 30 min; c: 3.50% bovine serum albumin (BSA) solution in water, room temperature, 60 min; d: DMSO/water (1/1), 40 °C, 60 min.; e: water, 40 °C, 30 min) and all tracers were obtained in 69−98% RCY (determined by radio-TLC).

**Scheme 31:**
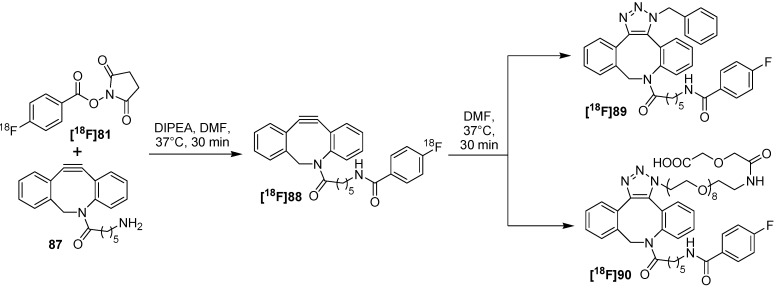
Labeling scheme for the labeling with **[^18^F]88**.

Furthermore, the work of Carpenter *et al.* was focused on the synthesis of a new ^18^F-labeled cyclooctyne building block [106] based on ADIBO which was recently synthesized by Popik *et al.* [107]. They used a 6-aminohexamido spacer and [^18^F]SFB **[^18^F]81** for the introduction of fluorine-18 into the building block. After purification via a C_18_-SPE cartridge, the ^18^F-labeled cyclooctyne **[^18^F]88** was obtained in 64 ± 15% RCY and > 80% RCP. As proof-of-principle, the following click-reaction was tested with benzyl azide and an azido-functionalized PEGylated acid at 37 °C in DMF to form the two regioisomers (ratio 1:1) **[^18^F]89** (74% RCY after 1 h) and **[^18^F]90** (64% after 2 h) (Scheme 31). HPLC-purified cyclooctyne **[^18^F]88** and triazoles **[^18^F]89** and **[^18^F]90** showed no decomposition or radiolysis over 6 h. Moreover, the stability of these products in PBS/saline buffer as well as in rat serum was tested. After 1 h, over 98% of intact radiotracer was found for both in rat serum. Additionally, formulations of both radiotracers were stable over 4 h in buffer.

In 2013, the same group evaluated an integrin α_v_β_6_-specific peptide and labeled it with fluorine-18 using this click-chemistry approach on the basis of the previously shown results. For that purpose, peptide N_3_-PEG_7_-A20FMDV2 (**91**) was yielded after azide-modification and preparation via solid-phase peptide synthesis [108]. Peptide **91** selectively targets the integrin α_v_β_6_-receptor which is located at the cell surface and has been identified as a prognostic indicator for several cancer entities [109,110].

**Scheme 32:**
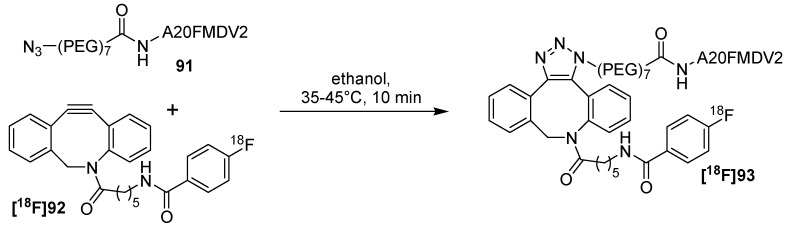
Labeling of N_3_-PEG_7_-A20FMDV2 (**91**) with [^18^F]FBA-C6-ADIBO **[^18^F]92**.

The click-reaction for the preparation of radiolabeled peptide **[^18^F]93** was performed in ethanol by treating peptide-precursor **91** with building block **[^18^F]92** for 10 min at 35–45°C (Scheme 32). After HPLC-purification, **[^18^F]93** was obtained as a regioisomeric mixture with 11.9% RCY (based on **[^18^F]92**) with > 99% RCP and with a A_S_ of 68 ± 25 GBq/μmol. The nonradioactive standard **[^19^F]93** was prepared from HPLC-purified [^19^F]FBA-C6-ADIBO **[^19^F]92** and N_3_-PEG_7_-A20FMDV2 (**91**) in 69% yield. Thus, equimolar amounts of the azide and the alkyne were combined in DMF and stirred at rt. Next, the *in vitro* stability of **[^18^F]93** was tested. After 60 min 94.6% of the tracer remained stable in rat serum, but after 2 h 80% of intact tracer was found. Cell binding studies of **[^18^F]93** revealed a strong α_v_β_6_-targeted binding (DX3puroβ6 cells, 15 min: 43.2% binding). *In vivo* studies using a mouse model demonstrated low binding to the DX3puroβ6-tumor (1 h: 0.47 ± 0.28% ID/g, 4 h: 0.14 ± 0.09% ID/g) and clearing from the bloodstream resulted via the renal and hepatobiliary routes. These results can be explained with the undesirable effects on pharmacokinetics which were expressed by the high lipophilicity and the large size of the labeling prosthetic group.

**Scheme 33:**
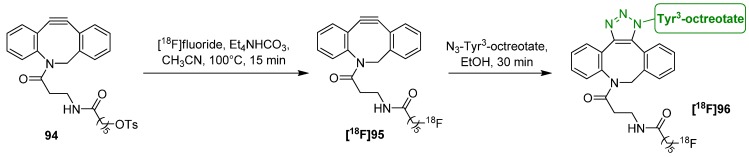
Preparation of [^18^F]ADIBO **[^18^F]95** and labeling of Tyr^3^-octreotate.

The next publication regards the preparation of [^18^F]ADIBO **[^18^F]95** by direct radiolabeling with [^18^F]fluoride [111]. Thus, ADIBO was modified with a 6-tosyloxyhexanoyl residue. Labeling was carried out via nucleophilic introduction of [^18^F]fluoride in the presence of Et_4_NHCO_3_ in acetonitrile at 100 °C for 15 min. [^18^F]ADIBO **[^18^F]95** was obtained in a RCY of 65%. Subsequent radiolabeling of an azide-functionalized Tyr^3^-octreotate (TATE) peptide was accomplished in ethanol. More than 95% of **[^18^F]95** was converted into **[^18^F]96** after 30 min (Scheme 33).

**Scheme 34:**
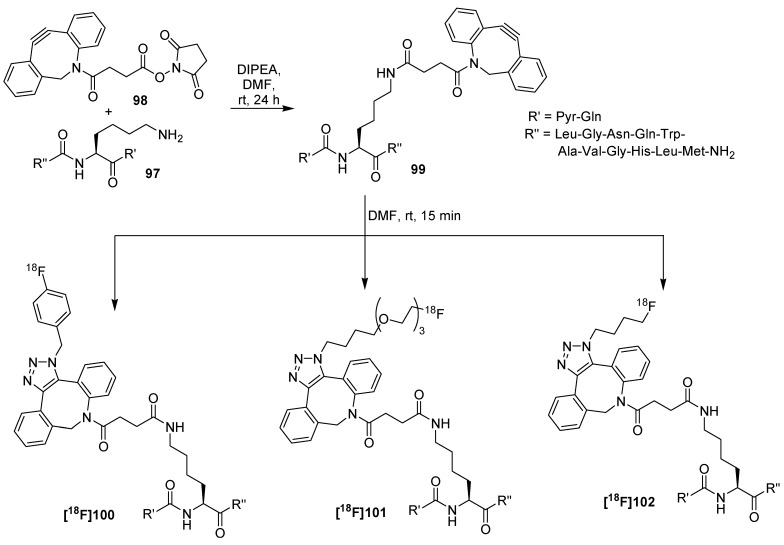
Preparation of Aza-DBCO-bombesin **99** and radiolabeling with three ^18^F-building blocks.

All following works used a change of functionalities to ^18^F-labeled azides which underwent a click-reaction with different cyclooctynes connected to the bioactive molecule of interest. The advantage of labeled azides is their versatile applicability. They can be used for Cu-mediated click-reaction, the variants of the Staudinger Ligation and for copper-free click reaction with cyclooctyne. To demonstrate the versatility, the group of Campbell-Verduyn *et al.* [112] modified lys[3]-bombesin **97** with aza-dibenzocyclooctyne **98** which was previously functionalized with a succinimidyl ester. Resulting peptide **99** was then reacted with three different fluorine-18-containing azides at room temperature for 15 min in DMF with radiochemical yields of 19−37% (Scheme 34). The binding affinities of these labeled bombesin-derivatives to gastrin-releasing peptide receptors were determined using human PC3 prostate cancer cells which revealed a high affinity despite the modifications (IC_50_ values: 29 nM to 40 nM).

**Scheme 35:**
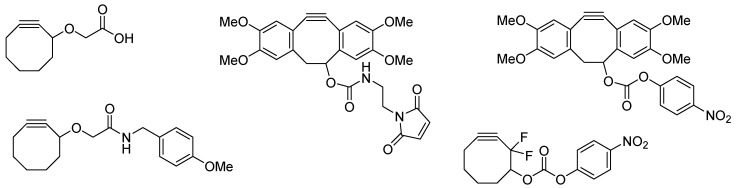
Cyclooctyne moieties for the radiolabeling with [^18^F]fluoroethylazide (**[^18^F]33**).

In 2012, Evans and co-workers demonstrated that CFC reactions can be carried out between [^18^F]fluoroethylazide (**[^18^F]33**) and five derivatives with different cyclooctyne scaffolds as pointed out in Scheme 35 [113]. The process of radiolabeling was optimized. Highest RCYs were obtained when using acetonitrile as solvent, a reaction time of 15 min, and a temperature of 90 °C. In addition, the biological behavior of **[^18^F]33** was analyzed using BALB/c nude mice.

**Scheme 36:**
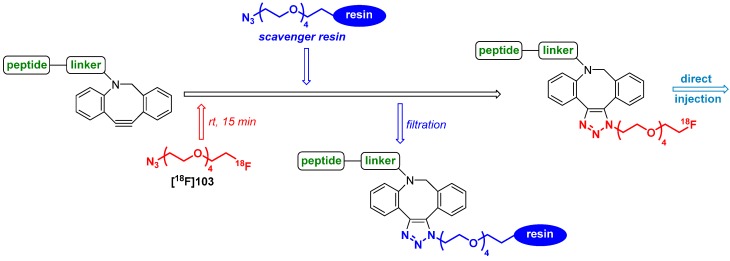
Overview of the general labeling and purification procedure.

The group of Kim *et al.* presented a protocol for the radiolabeling of peptides with fluorine-18 under physiological reaction conditions [114]. Four fluorine-18-labeled biologically active peptides for tumor targeting such as cyclic Arg-Gly-Asp (cRGD) peptide, bombesin (BBN), c-Met binding peptide (cMBP) and apoptosis targeting peptide (ApoPep) were employed for this purpose. Advantageously, the resulting ^18^F-peptides were provided as direct injectable solutions without any HPLC purification and/or formulation processes due to the application of a novel azide-functionalized scavanger resin after the labeling procedure. The remaining cyclooctyne precursor was cached with the azide-functionalized resin for purification purposes; the overview is given in Scheme 36.

**Scheme 37:**
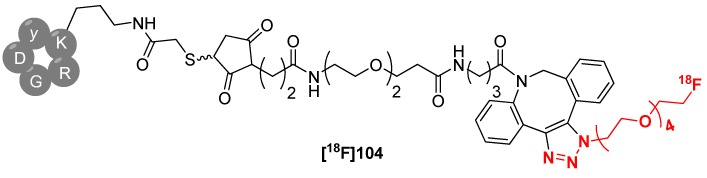
cRGD-ADIBOT-^18^F** [^18^F]104**.

In the first step, the desired peptides (cyclic Arg-Gly-Asp (cRGD) peptide, bombesin (BBN), c-Met binding peptide (cMBP) and apoptosis targeting peptide (ApoPep)) were modified with the cyclooctyne moiety. The next step involved the radiolabeling with **[^18^F]103** in an ethanol/water mixture (v/v = 1/1) at ambient temperature for 15 min. Afterwards, the azide-functionalized scavenger resin was added and the mixture maintained for 20 min to allow the remaining ADIBO-peptide to conjugate with the resin. After filtration and washing of the resin with PBS solution, all peptides were produced within approx. 35 min total reaction time in 92% RCY (d.c. for **[^18^F]104**) with > 98% of RCP as a direct injectable solution for further studies without any HPLC purification. cRGD-ADIBOT-^18^F **[^18^F]104** was shown as a representative example in Scheme 37.

## 5. Staudinger Ligation

**Scheme 38:**
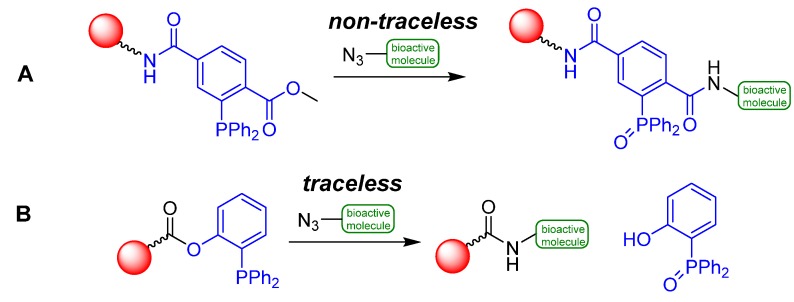
Variants of the Staudinger Ligation.

In 1919, Hermann Staudinger and Jules Meyer investigated the reaction of organic azides with phosphane derivatives [115]. Phosphane imines and elemental nitrogen were obtained from that reaction under absence of water. When water was added, the respective primary amines were yielded. This reaction is known as Staudinger reduction [116]. Conjugations with the respective phosphane imines are possible to execute using other electrophiles like carbonyl compounds instead of water. Based on these results, ligation reactions were developed which allow a bioorthogonal conjugation between azide functionalized molecules and phosphane species [117,118,119,120,121,122,123,124]. Two versions of this ligation type are known [125]. The non-traceless variant was introduced by Bertozzi *et al.* in the year 2000 [126]. Modified therephtalate compounds with a diphenylphosphanyl residue serve as fundament of this reaction. These modified phosphanes were reacted with organic azides and led to the desired ligation products after hydrolysis. Notably, the formed ligation product still contains the phosphane species which is oxidized during the reaction (Scheme 38: path **A**).

In the same year, the traceless variant of the Staudinger Ligation was independently and simultaneously developed by Raines and co-workers [127] and the group of Bertozzi [128]. In this case, organic azides were used as starting material as well. In contrast to the non-traceless ligation, phosphanes were introduced which are functionalized in the *ortho*-position relative to the phosphorus on one of the aromatic rings. The phosphane oxide residue which is formed during the reaction is excluded and both reaction partners are connected exclusively via a native amide (peptide) bond (Scheme 38: path **B**). Therefore, this reaction type is often adapted in peptide syntheses [129,130]. Both ligations proceed in a wide pH range using organic or aqueous solvents.

**Scheme 39:**
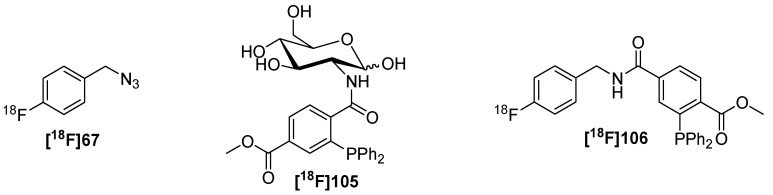
Building blocks for the radiolabeling using the non-traceless Staudinger Ligation.

The non-traceless Staudinger Ligation is seldom used for radiolabeling purposes with fluorine-18. To date, only one application is known. In 2006, the preparation of a fluorine-18 containing phosphane and the ligation to an azide containing carbohydrate was described [131] as shown in Scheme 39. In this case, the labeled glucose derivative served as an alternative for [^18^F]FDG and the application for the pretarget PET imaging of cancer was designated.

**Scheme 40:**
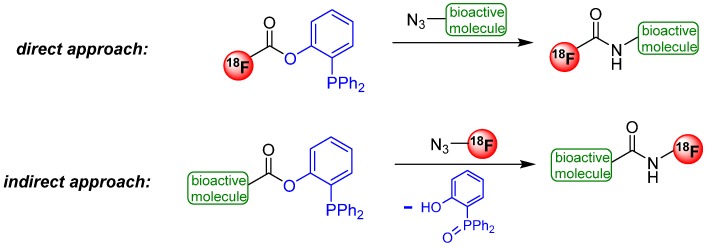
Direct and indirect approach of the traceless Staudinger Ligation.

In contrast to the non-traceless variant, several examples for the traceless Staudinger Ligation were published in the past. In general, two strategies were pointed out depending on the incorporation of fluorine-18 into the starting material. In the first case, phosphanes were evaluated containing the fluorine-18. For this purpose, biologically active molecules were modified with azide (direct approach). In the second case, the phosphane residue was introduced into the biologically active molecule and the fluorine-18 was connected to an azide functionalized molecule (indirect approach). Both ways are introduced in Scheme 40.

**Scheme 41:**
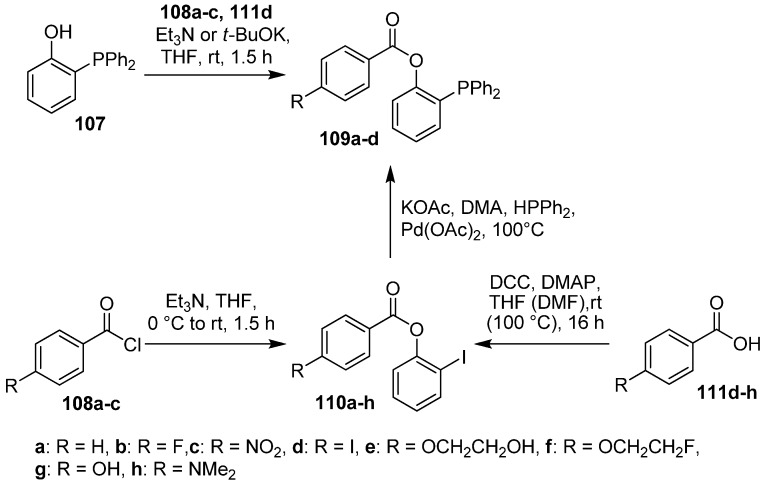
Preparation of various phosphane building blocks.

The direct approach was intensively investigated by Mamat and co-workers starting in 2009 [132]. For that purpose, phosphanes with various benzoate residues functionalized in the *para*-position were prepared [133]. Hence, two reaction paths were developed. First, the conventional esterification of phosphanol **107** with benzoyl chlorides **108a**–**c** in the presence of a base or via Steglich esterification (for **111d**) delivered the substituted phosphanes **109a**–**d** (yield: 38−92%). However, application of these reaction conditions sometimes led to low yields of the desired phosphanes or the formation of by-products. Due to this fact, functionalized 4-iodophenyl derivatives **110a-h** were applied as an alternative. Phosphanes **109a–h** were obtained in 58−89% yield from the Pd-catalyzed cross coupling of **110a–h** with diphenylphosphane (HPPh_2_); the findings are summarized in Scheme 41. This approach should serve as a possibility for the introduction of the 4-[^18^F]fluorobenzoate moiety (alternatively for [^18^F]SFB), but without success.

**Scheme 42:**
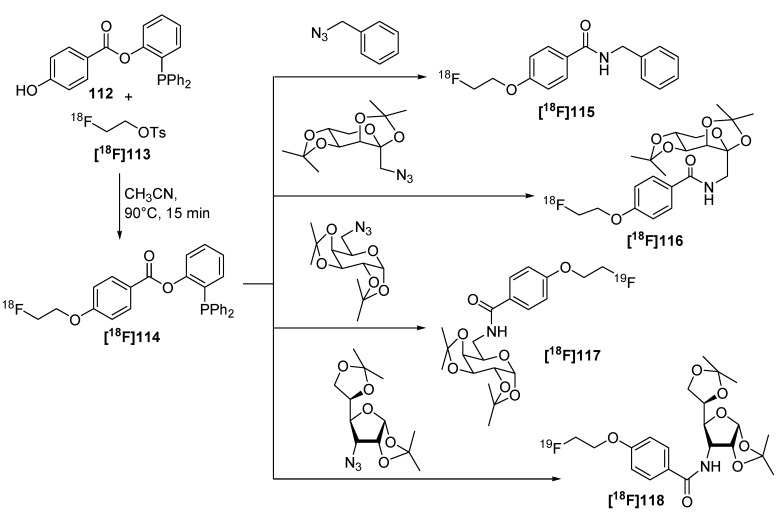
Radiofluorinations with **[^18^F]114** using the traceless Staudinger Ligation.

Later, a different labeling strategy was evaluated [134] based on 2-(diphenylphosphano)phenyl 4-hydroxybenzoate (**112**) which was reacted with 2-[^18^F]fluoroethyl tosylate (**[^18^F]113**) to yield the desired phosphane **[^18^F]114**. In the second step, the respective azide was added to yield the desired radiotracer. This reaction was carried out as a three-step/one-pot procedure starting from ethylene ditosylate as seen in Scheme 42. The phosphane precursor was added in a one-pot two-step procedure after the generation of **[^18^F]113** to yield the labeled phosphane species. Subsequently, the respective azido compound was added for labeling purposes within the Staudinger Ligation to yield **[^18^F]115-[^18^F]118** (17−21% RCY, d.c.).

**Scheme 43:**
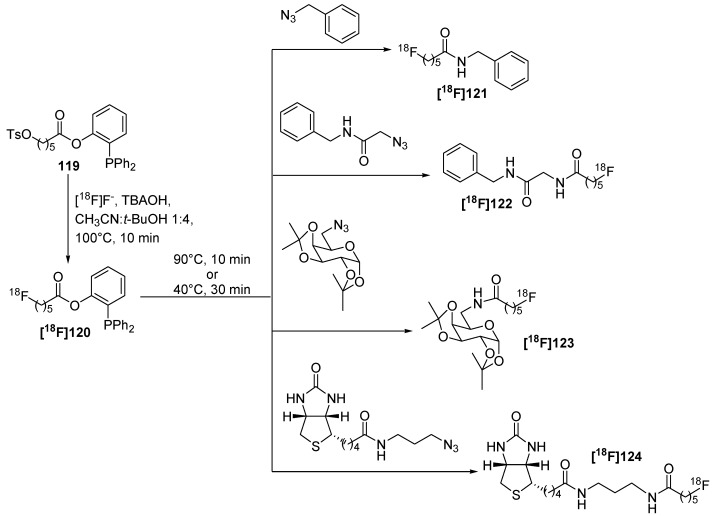
One pot preparation of phosphane building block **[^18^F]120** and subsequent radiolabeling of azide functionalized molecules using the traceless Staudinger Ligation.

Notably, a two-step sequence was required for the preparation of the ^18^F-containing phosphane which generally lowers the RCY as well as the A_S_ of the final tracers. For this purpose, a one-step approach for the preparation of fluorine-18 containing phosphane building blocks like **[^18^F]120** (RCY = 65%, d.c.) was developed based on aliphatic phosphanyl esters [135,136]. A chain length of five carbon atoms was chosen for precursor **119**. Different bases as well as fluorination agents and solvents were evaluated for optimization. The best results were obtained using [^18^F]fluoride in the presence of TBAOH which was used as base in a mixture of acetonitrile/*t*-BuOH (v/v = 1:4) [25] for 10 min at 100 °C using 21 mg of precursor **119**. Several model compounds **[^18^F]118-[^18^F]121** including a carbohydrate moiety and (+)-biotin were first azide functionalized and subsequently labeled using the traceless Staudinger Ligation (Scheme 43) with RCYs from 12 to 31% (d.c.).

**Scheme 44:**
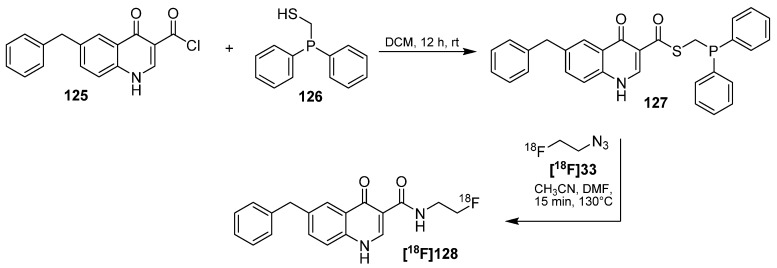
Preparation and radiolabeling of quinolone-based precursor **127**.

In 2010, Gaeta *et al.* reported the preparation and radiolabeling of GABA_A_ receptor binding 4-quinolones with K_i_ from 0.7 to 3.7 nM [137]. For this purpose, the bioactive site of molecule **125** was modified and (diphenylphosphino)methanethiol moiety **126** was introduced (indirect approach). For the radiolabeling, 2-[^18^F]fluoroethyl azide (**[^18^F]33**) was applied as the labeling building block. The following Staudinger Ligation was accomplished in a mixture of acetonitrile and DMF for 15 min at 130 °C and **[^18^F]128** was obtained after purification using preparative HPLC with 75% RCY (30–50 MBq, not d.c.) and a A_S_ = 0.9 GBq/μmol (radiochemical purity > 95%); see Scheme 44. Biological data showed that only a modest fraction of the compound crossed the blood–brain barrier, with peak uptake no greater than 0.12% of the injected dose (0.11% ID/g). *Ex vivo* autoradiographic analysis of prefrontal cortex, striatum and thalamus slices indicated significant differential binding of **[^18^F]128** consistent with the distribution of GABA_A_ receptors.

A second work was published in 2011 by the group of Gouverneur *et al.* [138] using a similar strategy with 2-[^18^F]fluoroethyl azide (**[^18^F]33**) as labeling agent and phosphane functionalized biologically active molecules **129–133** (indirect approach). In this case, various amino acid derivatives were labeled as shown in Table 2 [labeling conditions: (A) THF/H_2_O (4/1), 120 °C, 15 min; (B) DMF/H_2_O (6/1), 120 °C, 15 min].

**Table 2:** Scope of the labeling reaction under Staudinger conditions.

Starting material	Cond.	RCY^‡^	Product
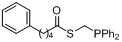 **129**	A	> 95%	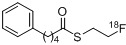 **134**
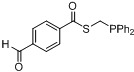 **130**	A	> 95%	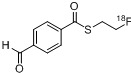 **135**
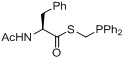 **131**	A B	> 95% > 95%	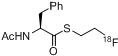 **136**
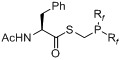 **132**	B	> 95%	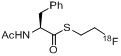 **136**
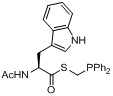 **133**	A	> 95%	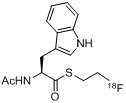 **137**

**^‡^** = conversion from 2-[^18^F]fluoroethyl azide (**[^18^F]33**), R_f_ = C_6_H_4_CH_2_CH_2_C_4_F_9_.

The purification step of this labeling reaction was improved using fluorous solid phase extraction (FSPE). For this purpose, derivative **132** was prepared with a fluorous tag [139] (C_4_F_9_ chain on the aromatic ring of the phosphane) attached onto the phosphino fragment. Adversely, this compound was faster oxidized to the corresponding phosphine than the non fluorous counterpart. Thus, the purification step of **132** was done under absence of air. The following Staudinger Ligation with 2-fluoroethyl azide using standard conditions led to the formation of **136** in 27% yield after purification of the reaction mixture using FSPE [140]. The decrease in isolated yield could be attributed to the propensity of the phosphane precursor **132** towards oxidation. The radiofluorination was performed in acetonitrile using [^18^F]^–^/K^+^/K 222 followed by FSPE to separate 2-[^18^F]fluoroethyl azide (**[^18^F]33**) (A_S_~10 GBq/mmol) from the excess fluorous precursor **132**, no distillation was necessary. Pleasingly, the labelling of both the non-fluorous and fluorous alanine precursor **131** and **132** with 2-[^18^F]fluoroethyl azide (**[^18^F]33**) in DMF–H_2_O (6 : 1) at 120°C for 15 min afforded **[^18^F]136** in an excellent RCY of >95% (entries 3–4, Table 2). The purification using FSPE for the labeling of **132** led to an improvement, but HPLC was still necessary to obtain an analytically pure sample due to the breakthrough of fluorous material into the fluorophobic eluted fraction.

## 6. Tetrazine-Click

**Scheme 45:**
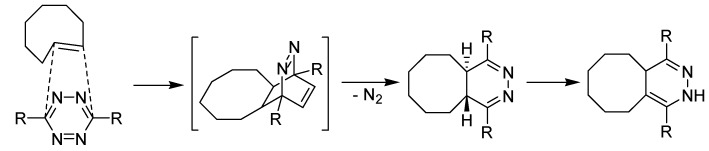
Proposed reaction mechanism of the tetrazine-click reaction.

Investigated first by the group of Fox and co-workers, the tetrazine ligation is a fast bioconjugation method based on inverse-electron-demand Diels−Alder reactions [141], a proposed mechanism is pointed out in Scheme 45. The main advantage of this kind of reaction involves the unusually fast reaction rates (*k*_2_ = 2,000 M^−1^·s^−1^, solvent MeOH/water: 9/1) without need for catalysis. A further advantage is the non-reversibility due to the loss of elemental nitrogen, in contrast to most other Diels-Alder reactions. These reactions tolerate a broad range of functionality and proceed with high yield in organic solvents, water, buffer, cell media, or lysate. This fast reactivity enables protein modification at low concentration. The resulting cycloocta[*d*]pyridazines were obtained as conjugation products from the cycloaddition of *s*-tetrazine and *trans*-cyclooctene derivatives. Mechanistically, the tetrazine ligation proceeds in two steps. An inverse-demand Diels-Alder reaction occurs followed by a retro-Diels-Alder reaction to eliminate nitrogen gas.

Based on computational work by Bach, *E*-cyclooctene has a highly twisted double bond resulting in a strain energy of 17.9 kcal/mol compared to *Z*-cyclooctenes or cyclooctane [142]. Based on these results, highly strained *trans*-derivatives were used as reactive dienophiles. Further, 3,6-diaryl-s-tetrazines serve as dienes which have been substituted in order to resist immediate reaction with water. The fact that the reaction is not only tolerant of water is also important especially for fast radiolabeling reactions. It has also been found that the rate increases in aqueous media. In addition, norbornenes were introduced as dienophiles at second order rates on the order of 1 M^−1^·s^−1^ in aqueous media. The labeling of live cells [143,144,145] or polymer coupling [146,147] were prominent examples of an application of this kind of conjugation reaction. Alongside, an elegant work concerning the radiolabeling of peptides using ^111^In-DOTA were published by Robillard [148] and a modular strategy for the preparation of ^64^Cu and ^89^Zr radiometalated antibodies was previously published [149].

**Scheme 46:**
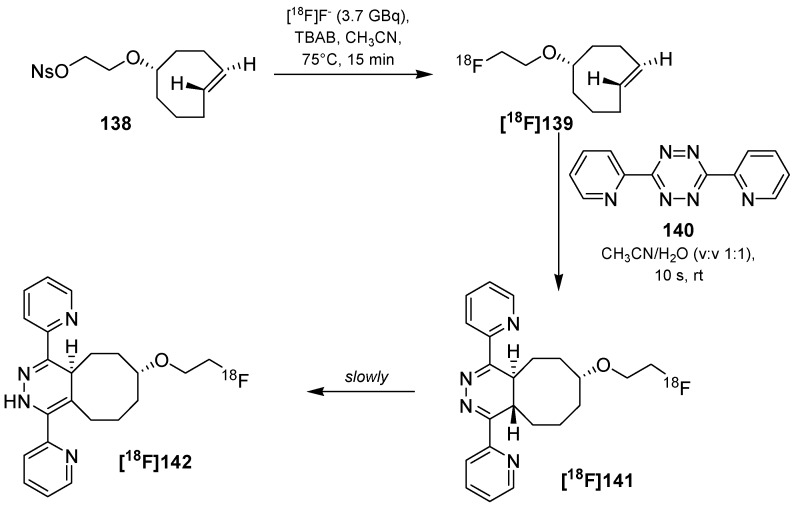
First example of the tetrazine-click reaction.

One of the first studies to apply the tetrazine-click reaction for radiofluorinations was achieved in the year 2010 by the group of Fox and co-workers (Scheme 46) [150]. First, they tried to label tetrazine derivatives with fluorine-18 for a conjugation with the *trans*-cyclooctene containing bioactive molecule. The instability of the tetrazines (RCY~1%) prompted the group to change the functionalities. Thus, [^18^F]TCO **[^18^F]139** as fluorine-18-containing cyclooctene derivative was prepared (RCY 71%) from the respective nosylate precursor **138** in a reaction time of 15 min. **[^18^F]139** was then used for the conjugation with the tetrazine containing sample molecule **140** within 10 s of mixing at ambient temperature with a RCY > 98%.

**Scheme 47:**
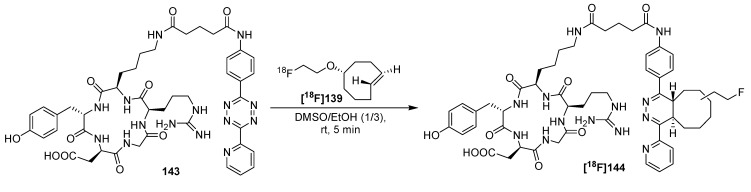
Labeling of tetrazine-modified RGD peptide **143** using **[^18^F]139**.

Based on these results, a cyclic RGD peptide was developed as an α_v_β_3_ targeted PET tracer [151]. Tetrazine-RGD conjugate **143** was prepared as the precursor by treatment of c(RGDyK) with a NHS-ester. Radiolabeling was done with [^18^F]TCO **[^18^F]139** at ambient temperature for 5 min in a mixture of DMSO and EtOH (1/3). [^18^F]RGD-tracer **[^18^F]144** was obtained in >95% RCY (based on **[^18^F]139**), see Scheme 47. microPET analyses using a U87MG xenograft model showed a tumor uptake of 4.4%ID/g after 1 h. Blocking experiments revealed a specific binding to integrin α_v_β_3_.

Next, the tetrazine residue was functionalized with a maleimide moiety for the radiolabeling of other cyclic RGD peptides without lysine residue [152]. Two strategies were evaluated for that purpose. In the first, the tetrazine-maleimide linker was conjugated with a cysteine-modified RGD peptide and finally labeled with [^18^F]TCO **[^18^F]139**. The labeled peptide was obtained in > 95% RCP with a A_S_ of approx. 111 to 222 GBq/µmol. To compare the efficiency of this ligation type, [^18^F]TCO **[^18^F]139** was directly reacted with the maleimide functionalized tetrazine. The original RGD peptide was then treated with this conjugate; however, this reaction proceeded within 20 min whereas the first labeling way took only 5 min. In addition, a higher peptide concentration was mandatory for the second way. MicroPET studies of U87MG tumor-bearing mice showed an uptake of ^18^F-RGD **[^18^F]144** of approx. 2% ID/g in the tumor which is comparable with the previous findings of this group.

**Scheme 48:**
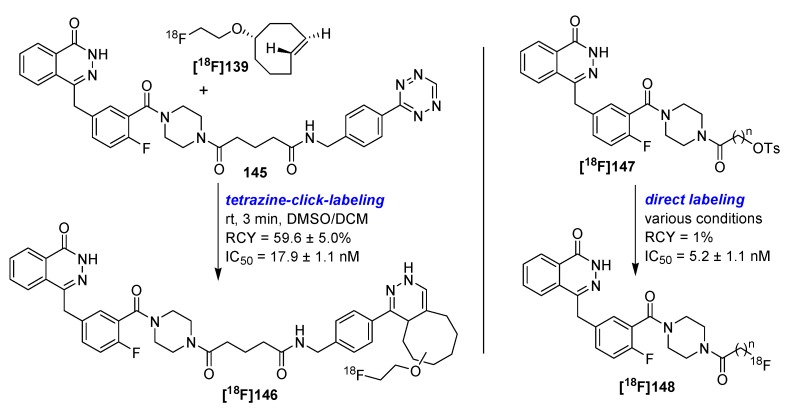
Comparison between conventional and tetrazine-click labeling.

Next, based on previous work [153], the syntheses and *in vivo* imaging of two ^18^F-labeled PARP1 inhibitors were presented by Reiner *et al.* in 2011 [154,155]. In the first publication, they compared the conventional direct nucleophilic introduction of [^18^F]fluoride with the click-labeling using [^18^F]TCO **[^18^F]139**. For this purpose, a fully automated synthesis (41 min) of [^18^F]TCO **[^18^F]139** was developed which yielded the building block in 44.7 ± 7.8% RCY (d.c., > 93% RCP, conditions: [^18^F]fluoride, *n-*Bu_4_NHCO_3_, 90 °C). Subsequent radiofluorination with tetrazine **145** was accomplished in a DMSO/DCM solution for 3 min. **[^18^F]146** was provided in 59.6 ± 5.0% RCY (d.c., n = 3, RCP > 96%) after HPLC purification. In contrast, the direct radiofluorination of **147** delivered **[^18^F]148** in only 1% RCY. Next, the target affinity of both tracers was studied. Thus, a colorimetric assay was employed to measure PARP1 activity with the result that **[^18^F]148** had an IC_50_ of 5.2 ± 1.1 nM whereas the clicked product **[^18^F]146** had an IC_50_ of 17.9 ± 1.1 nM. This results show impressively the dependence of the introduced moiety on the affinity of a molecule (Scheme 48).

**Scheme 49:**
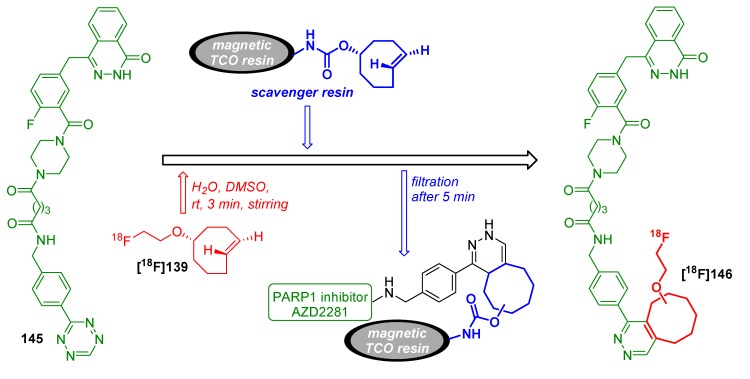
Overview of the general labeling and purification procedure.

An improvement was done regarding the purification of the resulting radiotracer **[^18^F]146** from the precursor **145** [156]. A magnetic TCO resin was developed and used for a subsequent incubation after the radiolabeling step. The following separation was done using a magnet. An overview was pointed out in Scheme 49. This method was applied to successfully remove the excess of precursor **145** and to avoid lengthy HPLC purifications. After separation of the magnetic resin, the radiotracer **[^18^F]146** was obtained in 92 ± 0.4% RCY (d.c.).

**Scheme 50:**
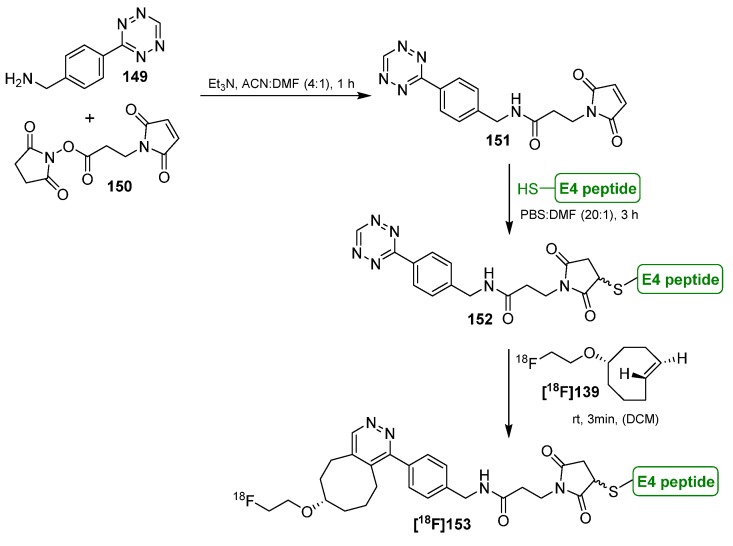
Radiolabeling of E4 peptide **[^18^F]153** using tetrazine-click.

In 2012, an efficient radiofluorination strategy regarding exendin-4 analogues of imaging of beta cells was described (Scheme 50) [157]. For this purpose, (*E*)-5-(2-[^18^F]fluoroethoxy)cyclooct-1-ene **([^18^F]139)** was used on the one hand and aminobenzyl tetrazine **149** was functionalized with maleimide **150** for a conjugation with SH-groups of peptides like cysteine. Therefore, a natural occurring lysine at position C12 was exchanged for a cysteine in an exendin-4-affine peptide (E4_C12_). Next, E4_C12_ peptide was modified with the tetrazine containing cross linker **151** to give E4_C12Tz_ **152**. Similar to the above mentioned techniques, E4_C12Tz_ **152** was incubated with [^18^F]TCO **[^18^F]139** at rt for 20 min and the labeled peptide **[^18^F]153** was obtained with > 94% RCP after separation using the aforepublished magnetic scavenger resin.

## 7. Miscellaneous

**Scheme 51:**
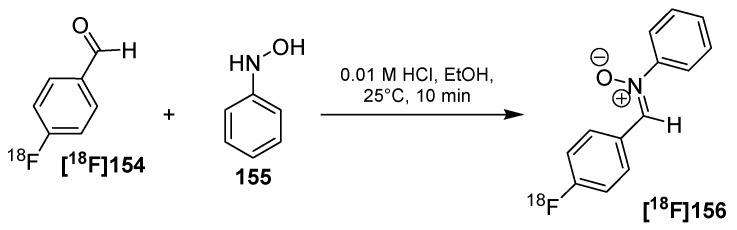
Preparation of C-(4-[^18^F]fluorophenyl)-*N*-phenyl nitrone (**[^18^F]156**).

In addition to the classical CuAAC, the 1,3-dipolar cycloaddition of nitrones with alkenes which led to isoxazolidines or further to isoxazoles belongs to the [3+2]-dipolar cycloadditions and was evaluated for radiofluorination purposes by the group of Zlatopolsky *et al.* in 2012 [158]. Mild reaction conditions, regioselectivity, absence of protection groups, acceptable yields and metabolic stability of the formed isoxazoles are the main advantages. Both starting materials are stable under a wide range of conditions (pH, temperature, microwave irradiation, reagents) [159] and this reaction can be carried out in different solvents, including aqueous media [160,161]. Of high importance is the absence of metal catalysts like Cu(I) in contrast to the classical CuAAC. In the first sample reaction, the fluorine-18 containing nitrone was simply prepared from the appropriate aldehyde **[^18^F]154** and *N*-substituted hydroxyl amines like **155**. Based on this strategy, the resulting labeling building block **[^18^F]154** was prepared in 74% RCY after 10 min reaction time at ambient temperature (Scheme 51).

**Scheme 52:**
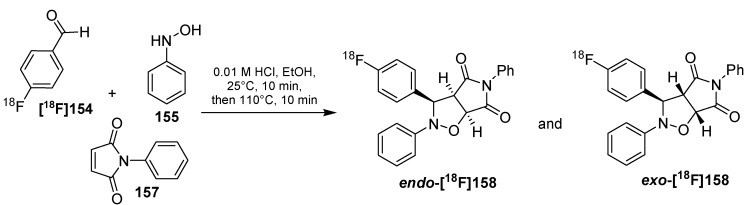
One-pot radiolabeling of model compound **157**.

The next step includes the radiolabeling of three sample molecule. For this purpose, these molecules were modified with a maleimide residue. The subsequent labeling step was carried out in a one-pot synthesis starting from 4-[^18^F]benzaldehyde (**[^18^F]154**), phenyl hydroxylamine (**155**) and the respective malinide species in RCYs > 80% (Scheme 52). The respective ^18^F-labeled diastereomers were formed as *endo*- and *exo*-isomers (ratio approx. 2/1 = *endo*/*exo*) in all cases and were easy separable.

**Scheme 53:**
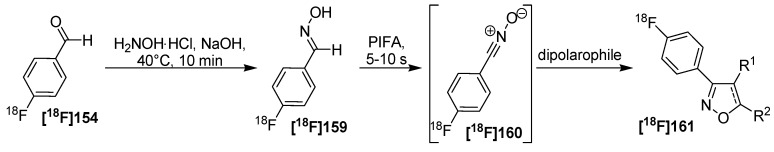
Overview of the modified reaction.

A further improvement of the labeling reaction was done in the same year [162]. In contrast to the first approach, hydroxylamine (as its HCl salt) was used instead of *N*-functionalized derivatives for the preparation of 4-[^18^F]benzonitrile oxide **[^18^F]159** as ^18^F-containing building block. **[^18^F]159** was obtained in 92% RCY with >90% RCP after 10 min in aqueous methanol at 40 °C from [^18^F]benzaldehyde **[^18^F]154** and hydroxylamine. For radiofluorination purposes, **[^18^F]159** was treated with phenyl iodine bis(trifluoroacetate) (PIFA) for 5–10 s. Afterwards, different dipolarophiles were chosen and even less reactive derivatives like styrene or 4-pentyn-1-ol showed high yields of the cycloaddition product (Scheme 53).

**Scheme 54:**
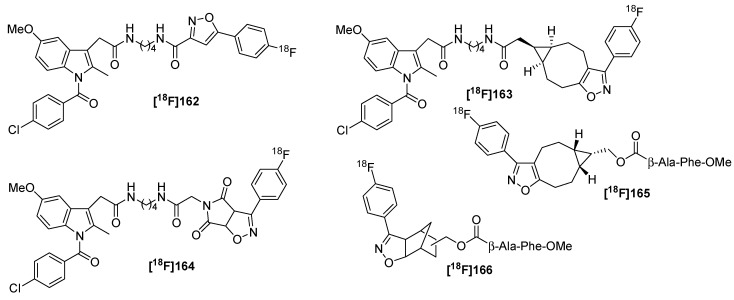
Radiolabeled indomethazin derivatives.

Using this reaction, three fluorine-18 labeled indomethacin derivatives **[^18^F]162-[^18^F]164** were prepared to enable visualization of the COX-2 expression in inflammation of cancer. For this purpose, the indomethacine scaffold was functionalized with maleimide, propyne and cyclooctyne. The highest results were obtained with 81% RCY using the cyclooctyne derivative. Results are shown in Scheme 54.

The last experiment contained the labeling of a dipeptide (β-Ala-Phe-OMe) either bound to the cyclooctyne residue or bound to the norbornene moiety. Both labeled peptides **[^18^F]165** and **[^18^F]166** are pointed out in Scheme 54. 

## 8. Conclusions

This review has summarized the applications and the scope of bioorthogonal click reactions for the introduction of fluorine-18 in the field of radiopharmacy. This kind of reaction has become a very important tool, especially for the radiofluorination of high molecular weight pharmacologically relevant molecules like peptides or proteins but also for the radiofluorination of small compounds of interest. For labeling purposes, fast and high yielding reactions are required and strategies for a convenient purification in an acceptable time span. 

**Table 5:** Reaction conditions and yields of selected ^18^F-containing building blocks.

Building block	RCY [%]	RCP [%]	t [min]	A_S_ [GBq/µmol]	Synthesis procedure	Literature
[^18^F]SFB	25–38	>95	70	n.d.	automatically	[163]
[^18^F]FBAM	29	94–98	40	13–17	automatically	[164]
[^18^F]AFP **[^18^F]47**	25–50	>97	40	3–6	automatically	[78]
[^18^F]BFP **[^18^F]46**	25–50	>98	40	5–9	automatically	[78]
[^18^F]Fluorobutyne **[^18^F]2a**	36	>98	40	n.d.	manually	[48]
[^18^F]Fluoropentyne **[^18^F]2b**	51	>99	40	n.d.	automatically	[50]
[^18^F]FSi-based azide **[^18^F]13**	54	>95	20	n.d.	manually	[51]
[^18^F]PEG_3_-alkyne **[^18^F]23**	57	>99	40	n.d.	automatic	[54]
[^18^F]PEG_3_-azide **[^18^F]27**	62	>99	40	n.d.	manually	[55,56]
[^18^F]FEA **[^18^F]33**	55	>99	40	n.d.	automatically	e.g., [57,58]
[^18^F]FDG-β-Az **[^18^F]78**	71	n.d.	30	n.d.	manually	[100]
[^18^F]F-SA **[^18^F]51**	32	>95	80	120–570	automatically	[81,82,83]
[^18^F]ArBF_3_^-^ **[^18^F]72**	50		70	555	manually	[95,96,97]
[^18^F]Fluoroethynylpyridine **[^18^F]59**	28	>98	35	n.d.	manually	[87]
[^18^F]FB-DBCO **[^18^F]82**	85 (from SFB)	95	60 (from SFB)	n.d.	manually	[105]
[^18^F]ADIBO **[^18^F]47**	73–95	95	55	3.8	manually	[111]
[^18^F]FHP **[^18^F]120**	65	n.d.	10	n.d.	manually	[135]
[^18^F]TCO **[^18^F]139**	46	>94	102	n.d.	automatically	[157]

After considering all results which were obtained in the last years a *guideline for the choice of building blocks* could be generated: first, a building block should be azide-functionalized for universal applicability and to prevent side-reactions like the Glaser coupling. This is important especially for radiolabeling purposes with alkyne functionalized building blocks using the CuAAC. In addition, when utilizing the CuAAC the use of Cu(I) stabilizing agents like TBTA, BTTAA and bathophenanthroline is crucial and should be at least 1.1 eq to the Cu-source. Second, the building blocks should contain ^18^F-propyl, ^18^F-ethyl or ^18^F-aryl groups for a higher metabolic stability. Third, these blocks should be directly radiolabeled by fluorine-18 for shorter preparation times and higher RCY and A_S_. Further, glycosylations or PEGylations of these building blocks enhance the *in vivo* availability of the corresponding radiolabeled bioactive molecule. An overview of building blocks is given in Table 5.

Moreover, it is necessary to separate and deplete catalysts like the copper species completely when using the Huisgen-click. This is important especially for *in vivo* applications due to the formation of complexes of Cu with the respective high molecular weight bioactive molecule during the labeling procedure. In this case and due to the toxic properties of copper species *in vivo*, copper-free variants should be applied. Thus, the CuAAC should be more preferred rather for small compounds. Last but not least, the non-radioactive reference building blocks as well as the precursors should be conveniently prepared from inexpensive chemicals.

Tetrazine-click as well as the Cu-free variant of the Huisgen cycloaddition show the best results in terms of the labeling time. This is important, especially for the labeling of high molecular weight compounds. Moreover, the removal of Cu or other catalysts is not needed. The size of the building block and the formation of regioisomers is a drawback, but the higher the molar mass of the molecule to be labeled the smaller is the influence of the building block in most of the cases. 

The use of cartridges or scavenger resins for a fast separation and purification is a fundamental topic. This allows labeling reaction with high yields, high radiochemical purity and high specific activity in a very short time. A high hydrophilicity of the bioorthogonal building blocks should be considered for the radiolabeling of proteins and antibodies.
